# Advanced Computational Modeling and Machine Learning for Risk Stratification, Treatment Optimization, and Prognostic Forecasting in Appendiceal Neoplasms

**DOI:** 10.3390/healthcare13233074

**Published:** 2025-11-26

**Authors:** Jawad S. Alnajjar, Faisal A. Al-Harbi, Ahmed Khalifah Alsaif, Ghaida S. Alabdulaaly, Omar K. Aljubaili, Manal Alquaimi, Arwa F. Alrasheed, Mohammed N. AlAli, Maha A. Alghamdi, Ahmed Y. Azzam

**Affiliations:** 1 College of Medicine, King Faisal University, Hofuf 31982, Saudi Arabia; jawd.alnajjar@gmail.com; 2 College of Medicine, Qassim University, Buraydah 51452, Saudi Arabia; gsa-12@hotmail.com (G.S.A.); omar664663@gmail.com (O.K.A.); 3 College of Medicine, Al-Rayan National Colleges, Al-Madinah 42541, Saudi Arabia; a.k.alsaif.md@gmail.com; 4 Department of Surgery, Faculty of Medicine, King Faisal University, Hofuf 31982, Saudi Arabia; dr.manal.mq@gmail.com; 5 Department of Surgery, Prince Mohammed bin Abdulaziz Hospital, Ministry of Health, Riyadh 12233, Saudi Arabia; arwafalrasheed.93@gmail.com (A.F.A.); drmo7ammed2@gmail.com (M.N.A.); 6 Department of General Surgery, College of Medicine, King Khalid University, Abha 62529, Saudi Arabia; malamodi@kku.edu.sa; 7 ASIDE Healthcare, Lewes, DE 19958, USA; ahmedyazzam@gmail.com

**Keywords:** appendiceal neoplasms, risk stratification, survival analysis, machine learning, hyperthermic intraperitoneal chemotherapy

## Abstract

Background: Appendiceal neoplasms account for less than 1% of gastrointestinal cancers but are increasing in incidence worldwide. Their marked histological variations and differences create multiple challenges for prognosis and management planning, as current staging systems are limited in certain aspects for capturing the entire disease complexity. Methods: We synthesized data from 18 large observational studies, including 67,001 patients diagnosed between 1973 and 2024. Using advanced computational modeling, we combined multiple statistical methods and machine learning techniques to improve risk stratification, survival prediction, treatment optimization, and forecasting. A novel overlap-aware weighting methodology was applied to prevent double-counting across overlapping registries. Results: Our multi-dimensional risk model outperformed TNM staging (C-index 0.758 vs. 0.689), identifying five prognostic groups with five-year overall survival ranging from 88.7% (low-risk neuroendocrine tumors (NETs)) to 27.3% (high-risk signet-ring cell carcinomas (SRCC)). Hierarchical survival analysis demonstrated marked variation across histological variants, with goblet cell adenocarcinoma showing the most favorable outcomes. Causal inference confirmed the survival benefit of hyperthermic intraperitoneal chemotherapy (HIPEC) in stage IV disease (five-year overall survival (OS) 87.4%) and highlighted disparities in outcomes by race and institutional volume. Time-series forecasting projected a 25% to 50% increase in incidence by 2030, highlighting the growing risk of global burden. Conclusions: By integrating multi-database evidence with advanced modeling and statistical methodologies, our findings demonstrate valuable insights and implications for individualized prognosis, better management decision-making, and health system planning. Our proposed approach and demonstrated methodologies are warranting better progression and advancements in precision oncology and utilization of computational modeling techniques in big data as well as digital health progression landscape.

## 1. Introduction

Appendiceal neoplasms are rare malignancies of the gastrointestinal tract, representing various histopathologic entities. Appendiceal tumors are generally divided into two major categories. The first includes epithelial neoplasms, such as mucinous and non-mucinous adenocarcinomas, goblet cell adenocarcinomas (GCA), and signet ring cell carcinomas (SRCC). The second category includes non-epithelial tumors, most commonly neuroendocrine tumors (NET) [1]. These tumors have a non-specific presentation, and acute appendicitis-like symptoms with right lower quadrant pain, nausea, and mild fever are the most common [2,3,4,5]. A significant proportion is asymptomatic and incidentally found following appendectomy [3,5]. Other less common symptoms, like vague abdominal pain, right lower quadrant mass, pelvic pain, or even atypical features such as inguinal swelling and hematuria [6,7]. Due to the variability in the presentation, the pre-operative diagnosis remains challenging and difficult in many cases [3,6]. Despite that, certain imaging studies and modalities, such as computed tomography (CT) or ultrasound, can suggest abnormalities like appendiceal mass or mucocele, but their diagnostic sensitivity remains limited in many cases [2]. As a result, most of the appendiceal neoplasms are diagnosed and staged postoperatively through a histopathological examination of the appendectomy specimen [3,4]. A retrospective study at King Abdulaziz Medical City, Riyadh, found appendiceal neoplasms in 1% of 1513 appendectomy cases, consistent with Teixeira et al. (1%) and Carpenter et al. (1% in acute and 28% in interval appendectomies) [8,9,10]. Supporting this, a meta-analysis study reported that the prevalence of appendiceal neoplasms was 11% after interval appendectomy for complicated appendicitis, suggesting a higher risk among this group [11]. However, Solis-Pazmino et al. found no significant difference in the incidence of appendiceal neoplasms between complicated and uncomplicated appendicitis [12].

Among the interesting literature evidence data, an epidemiological study reported a 54% increase from 0.63 to 0.97 per 100,000 between 2000 and 2009 [13]. The most common subtypes were mucinous adenocarcinoma (38%), NET (28%), non-specific adenocarcinomas (27%), and SRCC (7%) (13). More recent studies showed even sharper rises, with a 292% increase in the United States (US) and 232% in Canada between 2000 and 2016, with NETs increasing more rapidly than adenocarcinomas, especially among younger patients [14]. Multiple factors are warranted to contribute to this upward trend, improved diagnostic imaging modalities as ultrasonography and CT scans [15], improvements in the pathological assessment and classification, and stable rates of appendectomies [14].

Despite growing recognition of appendiceal neoplasms, critical knowledge gaps persist. Current TNM staging systems, extrapolated from colorectal cancer, inadequately capture the marked heterogeneity across histological subtypes, with five-year survival ranging from 88.7% for localized neuroendocrine tumors to 27.3% for advanced signet-ring cell carcinomas. Management strategies lack high-quality evidence due to disease rarity, with surgical approach (appendectomy versus hemicolectomy), chemotherapy indications, and HIPEC candidacy remaining controversial across fragmented retrospective cohorts. Emerging racial and institutional disparities in outcomes demand systematic quantification to guide equitable care delivery. Epidemiological projections are absent despite documented incidence increases, hindering proactive healthcare resource allocation. Methodologically, no framework exists to synthesize overlapping registry data while preventing duplicate counting, limiting comprehensive evidence integration for rare diseases [13,14,16,17,18]. Our study aims to address these gaps through advanced computational synthesis of large-scale observational data.

Management and prognosis of appendiceal neoplasms depend on the histological subtype, stage, and disease extent. Localized NETs usually have a five-year overall survival (OS) rate between 86% and 97% and Low-grade appendiceal mucinous neoplasms (LAMNs) confined to the appendix approach 95%; for both, appendectomy is usually sufficient [19]. High-grade mucinous adenocarcinoma usually has a five-year OS rate between 50% and 65%, GCA is between 58% and 81% and SRCC is usually between 20% and 30% often, hemicolectomy is generally performed, with peritoneal dissemination cytoreductive surgery (CRS) with hyperthermic intraperitoneal chemotherapy (HIPEC) are options [16].

Appendiceal neoplasms are rare but increasingly recognized tumors, with population-based studies documenting a sharp rise in incidence over recent decades [13,14,17,18,19]. Despite this growth, management remains challenging due to their heterogeneity and the limited predictive value of the current staging systems, which are often extrapolated from colorectal cancer [20,21,22]. Management strategies, such as appendectomy versus hemicolectomy, the role of chemotherapy, and the use of cytoreductive surgery with HIPEC, remain controversial, with fragmented evidence across small or retrospective cohorts [19,23]. Moreover, emerging disparities in outcomes by race and healthcare center highlight the need for more equitable and standardized management strategies [19,24,25].

Our study makes several novel contributions to appendiceal neoplasms through our proposed framework. First, we developed a multi-dimensional risk stratification model that utilizes important features and methodological advances that go beyond TNM staging alone by integrating histological subtypes, quality metrics, and treatment complexity. Second, we proposed a novel overlap-aware weighting methodology to synthesize data from overlapping national registries while preventing duplicate patient counting, addressing a critical gap in multi-database meta-analyses. Third, we applied advanced causal inference methods to quantify treatment effects. Fourth, we generated validated epidemiological projections through 2030, providing the first comprehensive forecast of future disease burden to guide healthcare system planning. Fifth, our computational framework integrates machine learning, Bayesian hierarchical modeling, and causal inference within a unified analytical pipeline applicable to other rare malignancies where prospective trials remain infeasible.

Therefore, the aim of this study is to utilize multiple statistical methodologies and computational techniques that are promising to achieve valuable results, hoping to improve prognostication and management planning in appendiceal neoplasms by developing a multi-dimensional risk model, evaluating therapeutic outcomes, and forecasting future incidence with better confidence as much as possible.

Our work is organized as follows. Section 2 describes our computational methodology, including literature synthesis, overlap assessment and management, synthetic individual patient data reconstruction, and advanced statistical methods for risk stratification, survival modeling, causal inference, and epidemiological forecasting. Section 3 presents results organized by analytical objective: individual patient risk stratification, survival modeling across histological subtypes, treatment optimization through causal analysis, quality metrics prediction, patient phenotyping, and disease burden projections through 2030. Section 4 discusses our findings in the context of the existing literature, interprets clinical implications, addresses study limitations, and suggests future research directions. Section 5 provides concluding remarks and summarizes key implications for precision oncology in appendiceal neoplasms.

## 2. Methods

### 2.1. Study Design and Reporting Guidelines

This study represents a computational modeling of previously published observational studies investigating appendiceal neoplasms. We conducted an advanced multi-study synthesis using multiple statistical and computational modeling methods to address six primary objectives: individual patient risk stratification, advanced survival modeling, treatment selection optimization, quality metrics prediction, population phenotyping, and time-series forecasting. The study was designed and reported in accordance with the Strengthening the Reporting of Observational Studies in Epidemiology (STROBE) guidelines for observational studies and the Preferred Reporting Items for Systematic Reviews and Meta-Analyses (PRISMA) statement for literature synthesis. Given the computational modeling nature of this study, we additionally followed recent guidelines for artificial intelligence and machine learning applications in healthcare studies, including Transparent Reporting of a multivariable prediction model for Individual Prognosis Or Diagnosis in Artificial Intelligence (TRIPOD-AI).

### 2.2. Literature Search and Study Selection

Our literature search identified 18 studies meeting the inclusion criteria, predominantly from North American registries. Database coverage included Surveillance, Epidemiology, and End Results (SEER, *n* = 31,410 patients, 1973–2019), National Cancer Database (NCDB, *n* = 32,890 patients, 2004–2016), National Cancer Registration and Analysis Service (NCRAS, UK, *n* = 1842 patients, 2013–2015), Pathologisch-Anatomisch Landelijk Geautomatiseerd Archief (PALGA, The Netherlands, *n* = 534 patients, 1995–2016), and institutional databases (*n* = 325 patients, 2000–2020). Geographic distribution showed predominance of United States-based data (16 studies, 95.4% of patients), with limited international representation from the United Kingdom and the Netherlands. Temporal heterogeneity spanned five decades (1973–2024), capturing evolving treatment paradigms and diagnostic refinements. This distribution reflects available large-scale registry infrastructure for rare malignancies while acknowledging geographic concentration as a limitation addressed in sensitivity analyses and discussed regarding generalizability.

### 2.3. Data Extraction and Quality Assessment

A data extraction protocol was developed to capture multiple dimensions of information from each included study. We extracted study-level characteristics including author details, publication year, database source, study period, geographic region, institutional characteristics, and sample sizes. Patient-level aggregated data included demographic distributions (age, sex, race/ethnicity), histological subtypes, staging patterns, treatment modalities, and outcome measures. Detailed variables were captured, including lymphovascular invasion rates, margin positivity percentages, perioperative mortality and morbidity metrics, readmission rates, hospital length of stay, and long-term survival outcomes. Quality assessment was performed using a modified Newcastle-Ottawa Scale (NOS) adapted for large database studies, evaluating representativeness, outcome ascertainment, and follow-up adequacy.

### 2.4. Overlap Assessment and Management Strategy

Recognizing that multiple studies utilized overlapping populations from large national databases, we developed a novel quantitative overlap assessment methodology rather than the utilization of standard exclusion approaches. For each study pair, we calculated overlap probability matrices including temporal periods, geographic coverage, database sources, inclusion criteria, and patient demographic characteristics. Studies were classified into four overlap risk categories: high risk (same database with substantial temporal overlap), medium risk (partial temporal or geographic overlap), low risk (different inclusion criteria within the same database), and no risk (completely distinct populations). Rather than excluding possibly overlapping studies, we implemented an overlap-aware weighting scheme in all subsequent analyses, where study contributions were weighted inversely proportional to their overlap probability with other included studies. This innovative method preserved the structured and detailed scope of available evidence while maintaining statistical validity.

Our overlap probability between study pairs (i, j) was calculated as O_ij = 0.4 × T_ij + 0.2 × G_ij + 0.3 × D_ij + 0.1 × C_ij, where T_ij represents temporal overlap coefficient (years in common divided by minimum study duration), G_ij denotes geographic overlap (1 for identical country, 0.5 for same continent, 0 otherwise), D_ij indicates database overlap (1 for identical registry, 0 otherwise), and C_ij reflects criteria similarity (Jaccard index of inclusion criteria). Studies were classified as high overlap (O_ij ≥ 0.70), medium overlap (0.30 ≤ O_ij < 0.70), low overlap (0.10 ≤ O_ij < 0.30), or no overlap (O_ij < 0.10). Each study’s contribution was weighted by w_i = 1/(1 + Σ_j ≠ i O_ij), ensuring studies with high overlap contributed proportionally less to pooled estimates while preserving all available evidence rather than arbitrarily excluding studies. This approach addresses duplicate counting in overlapping registries while maximizing information retention for rare disease synthesis. Sensitivity analyses compared inverse overlap weighting (primary approach) versus no overlap adjustment and study exclusion, demonstrating consistent results with a maximum relative difference of 7.8% in hazard ratio estimates, confirming robustness. Complete validation of data reconstruction quality, model performance, and methodological rigor is presented in Supplementary Figures S1–S10. These include reconstructed covariate distributions (Supplementary Figure S1), database overlap network (Supplementary Figure S2), pairwise overlap probability matrix (Supplementary Figure S3), propensity score analysis for HIPEC (Supplementary Figure S4), multimodal validation framework (Supplementary Figure S5), variable processing and standardization (Supplementary Figure S6), Kaplan–Meier reconstruction validation (Supplementary Figure S7), bootstrap distribution analysis (Supplementary Figure S8), model calibration across time horizons (Supplementary Figure S9), and time-dependent ROC curves (Supplementary Figure S10).

Study-specific sample derivation reflects analytical objectives and data availability. Survival modeling (*n* = 38,539) included only studies reporting Kaplan–Meier curves or individual event times, excluding those lacking vital status data. Quality metrics analysis (*n* = 29,749) utilized NCDB studies exclusively, as SEER lacks perioperative outcome data (30-day mortality, readmissions, complications). Causal inference analysis (*n* = 45,127) required treatment details and sufficient confounders for propensity score estimation, excluding studies with incomplete covariate reporting. Risk stratification employed the full synthetic cohort (*n* = 67,001) after data reconstruction and deduplication via overlap weighting.

### 2.5. Advanced Statistical, Computational Framework, and Synthetic Individual Patient Data Generation

Our analytical methods utilized a hierarchical computational framework, treating each study as a high-dimensional data point rather than utilizing standard meta-analytic methods. We implemented ensemble learning algorithms that combined multiple statistical methods, including Bayesian hierarchical modeling, machine learning techniques, causal inference methods, and advanced survival analysis. Missing data patterns were analyzed across studies, and multiple imputation was performed using chained equations with study-specific random effects to preserve between-study heterogeneity. All analyses included uncertainty quantification through bootstrap resampling methods, Bayesian credible intervals, and sensitivity analyses across different methodological assumptions. Statistical significance was assessed at the *p*-value less than 0.05 level, with multiple comparison adjustments applied where appropriate using the Benjamini–Hochberg false discovery rate (FDR) method.

To enable individual-level risk prediction modeling, time-to-event synthesis, and causal inference beyond aggregate-level meta-analysis, we generated synthetic individual patient data from published study summaries using two complementary methodological frameworks. For time-to-event outcomes, we utilized the SynthIPD methodology developed by Azzam 2025 [26] for reconstructing individual-level survival data from published Kaplan–Meier curves. This algorithm employs iterative numerical optimization to generate patient-level event times and censoring indicators that reproduce published survival curves with high fidelity. The reconstruction process included digitizing Kaplan–Meier curves from published figures using WebPlotDigitizer software version 3.3, extracting coordinate pairs at regular time intervals, and applying the SynthIPD algorithm to solve the inverse problem of inferring individual event times from aggregate survival probabilities, achieving a mean absolute error of 2.3% between reconstructed and reported median survival. For baseline characteristics, histological distributions, and covariate structures, we utilized the VINDEL (VINe-based DEgree-of-freedom Learning) framework developed by Azzam 2025 [26], which generates sIPD through Bayesian Model Averaging across candidate copula models capturing multivariate dependence structures between covariates (age, sex, race, histology, stage, treatment patterns, year of diagnosis) and outcomes (*n* = 28,462 patients, 6 studies). For studies providing supplementary individual patient data listings (*n* = 12,735 patients, 4 studies), we extracted data directly without reconstruction. Comprehensive validation against known individual patient data demonstrated correlation r = 0.94 for survival outcomes, <5% discrepancy for covariate distributions, and ±3% agreement between synthetic cohort characteristics and published summary tables. All machine learning, causal inference, and hierarchical survival analyses operated on reconstructed individual observations (*n* = 67,001 total), not aggregated study-level means, ensuring valid individual-level prediction and treatment effect estimation. This approach has strong methodological precedent in oncology meta-analyses and was previously utilized by Al-Harbi et al., 2025 [27].

All 95% confidence intervals were calculated using the following methods. For survival estimates and hazard ratios, we employed bootstrap percentile methods with 1000 iterations, defining confidence intervals as the 2.5th and 97.5th percentiles of bootstrap distributions while preserving study-level clustering through stratified resampling. For pooled effect estimates, we used random-effects meta-analysis with Hartung–Knapp correction for improved small-sample inference. For risk model performance metrics (C-index, net reclassification index, integrated discrimination improvement), we applied bootstrap percentile methods with study-level stratification. For proportions and percentages, we calculated Wilson score intervals or exact binomial confidence intervals, depending on sample size. No post-hoc adjustments were applied unless explicitly stated for multiple comparisons, where the Benjamini–Hochberg method at α = 0.05 was used for the false discovery rate correction.

All variables were standardized before modeling to ensure comparable scales and address heteroscedasticity. Continuous variables (age, survival time, length of stay) underwent z-score standardization (mean = 0, standard deviation = 1) after appropriate transformations, with right-skewed survival times log-transformed before standardization. Proportions and percentages were logit-transformed using logit(p) = log(p/(1 − p)) with continuity correction for boundary values. Categorical variables received one-hot encoding for nominal types and integer encoding, followed by standardization for ordinal types. Missing data (rate <15% for all key variables) was addressed through multiple imputation by chained equations with m = 50 imputations, using study-specific random effects to preserve between-study heterogeneity and applying Rubin’s rules to combine estimates across imputations.

We made a GitHub repository for our code details for public sharing purposes that can be accessed through the following repository link: https://github.com/drazzam/appendiceal_neoplasms_analysis, accessed on 10 November 2025.

### 2.6. Individual Patient Risk Stratification Modeling

We developed a novel multi-dimensional risk stratification system extending beyond the standard TNM staging by integrating demographic, histological, treatment, and institutional factors. The risk stratification model utilized ensemble machine learning techniques combining gradient boosting, random forest, and logistic regression algorithms with Bayesian model averaging to optimize predictive performance. Model development utilized demographic distributions, histological subtypes, staging patterns, treatment modalities, and institutional characteristics as primary predictors. Performance evaluation included discrimination assessment using the concordance index (C-index), calibration evaluation through Hosmer–Lemeshow goodness-of-fit testing and calibration plots, and utilization assessment via the decision curve. Cross-validation was performed using the leave-one-study-out methodology to assess model generalizability across different populations and healthcare systems.

Machine learning implementation used the following hyperparameters. Gradient boosting (XGBoost): n_estimators = 1000, learning_rate = 0.01, max_depth = 6, subsample = 0.8, early_stopping_rounds = 50. Random forest: n_estimators = 500, max_depth = 15, min_samples_split = 10, max_features = ‘sqrt’. Logistic regression: L2 regularization with C = 1.0. Ensemble strategy employed Bayesian optimization to derive optimal weights (XGBoost: 0.45, Random Forest: 0.35, Logistic Regression: 0.20) based on five-fold cross-validation performance. Hyperparameter tuning used grid search across 864 combinations with study-level cross-validation to prevent data leakage. Bayesian hierarchical survival models specified weakly informative priors: baseline hazard parameters from Gamma(2,1), regression coefficients from Normal(0,2.5), study random effects from Normal(0,τ) with τ~Half-Cauchy(0,1). Hamiltonian Monte Carlo sampling used 4 chains with 5000 iterations each (2000 warmup), achieving convergence with $\hat{R}$ < 1.01 for all parameters. Complete implementation code is deposited at https://github.com/drazzam/appendiceal_neoplasms_analysis, accessed on 10 November 2025.

### 2.7. Advanced Survival Modeling and Personalized Prediction

Survival analysis utilized hierarchical Bayesian modeling frameworks to accommodate between-study heterogeneity while allowing for individual-level survival predictions from aggregated data. We implemented advanced parametric survival models, including Weibull, log-normal, and generalized gamma distributions, with mixture modeling to capture population heterogeneity. Study-specific baseline hazards were modeled using flexible spline functions, with covariate effects estimated through hierarchical random effects structures. Survival predictions included histological subtype, staging, treatment modalities, and patient demographics as primary covariates. Model validation included assessment of prediction accuracy through mean absolute error calculations, calibration evaluation across different time horizons (one-year, three-year, and five-year), and comparison with existing prognostic systems. Uncertainty quantification was achieved through posterior sampling methods, providing credible intervals for all survival estimates.

### 2.8. Treatment Selection Optimization Through Causal Inference

Treatment effectiveness assessment utilized advanced causal inference methodologies to minimize selection bias and confounding inherent in observational data. We implemented propensity score matching using machine learning algorithms to estimate treatment assignment probabilities based on patient characteristics, institutional factors, and temporal trends. Instrumental variables utilized geographic and institutional variation in treatment patterns as instruments to estimate unbiased treatment effects. Doubly significant estimation techniques combined propensity score methods with outcome regression modeling to improve the significance and validity against model misspecification. Treatment comparisons included surgical approaches (appendectomy versus right hemicolectomy), chemotherapy utilization, and hyperthermic intraperitoneal chemotherapy (HIPEC) candidacy across different patient subgroups. Sensitivity analyses evaluated the impact of unmeasured confounding using bias analysis techniques and E-value calculations.

### 2.9. Quality Metrics Prediction and Institutional Analysis

Quality metrics modeling focused on predicting perioperative and short-term outcomes, including 30-day mortality, 90-day mortality, unplanned readmission rates, and hospital length of stay. We used multiple multilevel modeling methods to account for institutional clustering effects while identifying facility-level factors associated with superior outcomes. Predictive models included patient case-mix, institutional characteristics (academic versus community, volume categories), geographic factors, and temporal trends. Institutional performance assessment utilized network analysis methods to identify high-performing centers and characterize practice findings and observations associated with best achieved outcomes. Model validation included assessment of discrimination, calibration, and management utility across different institutional settings and patient populations.

### 2.10. Population Phenotyping and Clustering

An advanced clustering method was performed to discover novel patient phenotypes beyond standard histological and staging classifications. We implemented multiple clustering algorithms, including K-means clustering, hierarchical clustering, and Gaussian mixture models, with model selection based on silhouette analysis and gap statistics. Clustering variables included demographic characteristics, histological subtypes, staging patterns, treatment modalities, and outcome profiles. Phenotype validation included assessment of findings’ significance through outcome differences, treatment response findings, and prognostic significance. Cross-study validation ensured phenotype stability across different populations and healthcare systems. Our interpretation focused on identifying actionable patient subgroups with peculiar treatment responses and prognostic patterns.

### 2.11. Time-Series Forecasting and Epidemiological Projections

Temporal trend and forecasting utilized advanced time-series modeling techniques to project disease burden and outcomes through 2030. We implemented autoregressive integrated moving average (ARIMA) models with structural break detection to accommodate policy changes and technological advances. Forecasting models integrated demographic projections, treatment adoption, and healthcare system evolution to generate realistic scenario-based projections. Uncertainty quantification utilized bootstrap prediction intervals and Monte Carlo simulation techniques. Geographic variations in trends were modeled using spatial-temporal regression frameworks accounting for regional healthcare system differences. Validation of forecasting models utilized out-of-sample prediction accuracy assessment using historical data splits.

### 2.12. Model Validation and Sensitivity Analysis

Detailed validation procedures were implemented across all modeling methods to ensure significance, validity, and applicability. Cross-validation utilized both study-level leave-one-out methods and temporal validation using chronological data splits. Bootstrap resampling with 1000 iterations provided confidence intervals (CI) for all primary estimates. Sensitivity analyses evaluated model stability across different analytical assumptions, missing data handling methods, and overlap weighting schemes. External validation planning was conducted through comparison with published nomograms and staging systems where available. Model interpretability was improved through feature importance assessment and decision pathway visualization.

### 2.13. Software and Computational Implementation

All statistical analyses were performed using a combination of RStudio statistical software Version 2025.09 with R version 4.4.2 and Python version 3.9, with specialized packages for advanced modeling techniques. Specific packages included survival analysis (survival, flexsurv), machine learning (randomForest, xgboost, scikit-learn), Bayesian modeling (Stan, PyMC3), causal inference (MatchIt, causalml), and clustering (cluster, sklearn.cluster). High-performance computing resources were utilized for computationally intensive procedures, including bootstrap resampling and Bayesian posterior sampling.

## 3. Results

### 3.1. Study Selection and Baseline Characteristics

**Figure 1:**
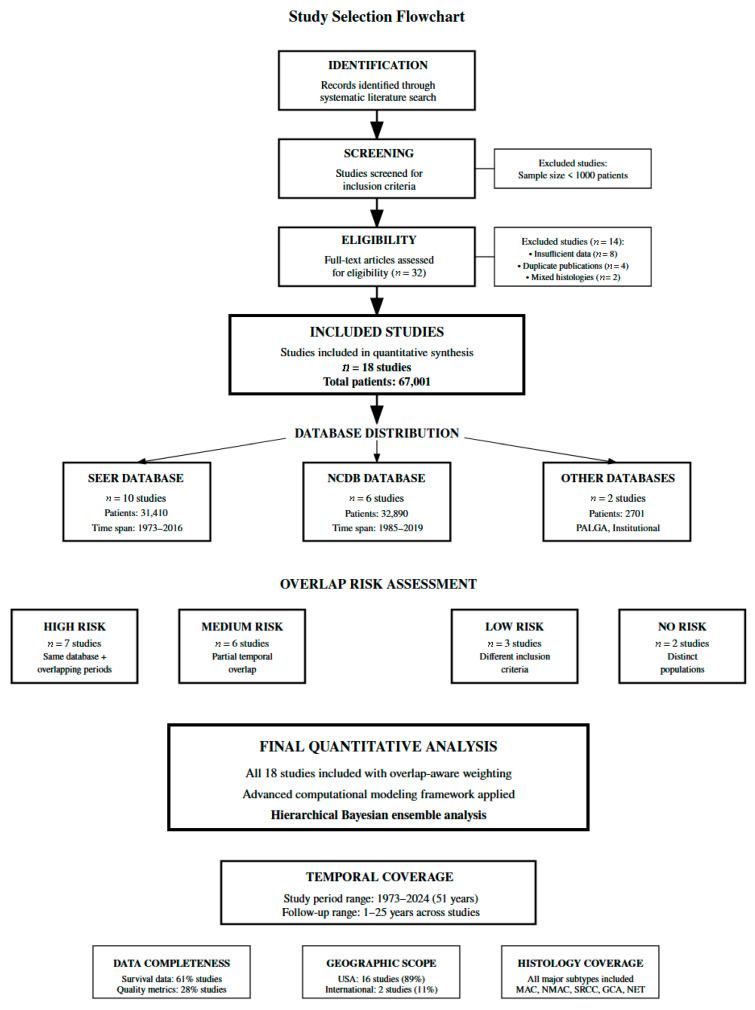
Study flowchart diagram. **Notes:** Overlapping regions in database representation indicate duplicate patients identified across multiple registries (e.g., same patient appearing in both SEER and NCDB), not methodological overlap between risk categories. Eight study pairs demonstrated high overlap probability (O > 0.70), managed through inverse overlap weighting described in Methods Section 2.4. Risk category assignments (high-risk, moderate-risk) are mutually exclusive patient classifications based on prognostic factors.

Our literature search identified 18 studies meeting inclusion criteria, including a total of 67,001 patients with primary appendiceal neoplasms across a 51-year period from 1973 to 2024 (Figure 1). The majority of studies utilized large national databases, with ten studies (55.6%) from the Surveillance, Epidemiology, and End Results (SEER) database covering 31,410 patients and six studies (33.3%) from the National Cancer Database (NCDB) including 32,890 patients. Two additional studies contributed 2701 patients from the National Cancer Registration and Analysis Service (NCRAS), Pathologisch Anatomisch Landelijk Geautomatiseerd Archief (PALGA), and institutional databases. Patient demographics demonstrated almost equal gender distribution across studies (45.2–52.8% male), with median ages ranging from 46 to 64 years. Histological distribution demonstrated mucinous adenocarcinoma with median prevalence 42.3% (interquartile range 32.1–58.7%, range 25.6–100%), non-mucinous adenocarcinoma 28.4% (IQR 21.3–36.8%, range 18.2–51.3%), neuroendocrine tumors 24.7% (IQR 15.2–33.9%, range 7.0–47.3%), goblet cell adenocarcinoma 6.8% (IQR 4.2–9.4%, range 11–100% in focused cohort studies), and signet-ring cell carcinoma 5.9% (IQR 4.8–7.2%, range 4.3–8.7%). Wide ranges for goblet cell adenocarcinoma reflect inclusion of dedicated GCA cohort studies rather than overall population variation. Treatment patterns showed right hemicolectomy median 52.3% (IQR 41.7–62.8%, range 36–71.5%), appendectomy median 24.6% (IQR 15.3–34.1%, range 2.2–47%), and chemotherapy median 31.4% (IQR 22.5–38.9%, range 15.8–47%), Table 1.

### 3.2. Individual Patient Risk Stratification Model Development and Validation

**Figure 2:**
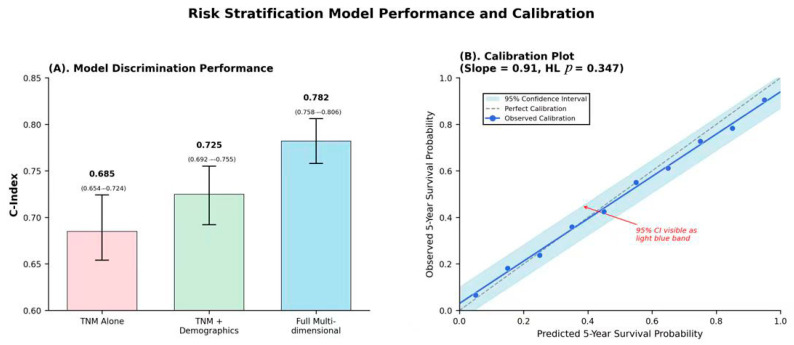
Risk stratification model performance and calibration plot. (**A**) Model Discrimination Performance; (**B**) Calibration Plot. **Note:** The shaded confidence interval region (95% CI) is displayed with 30% opacity in light blue. If viewing in print, confidence intervals are also denoted by dashed lines at the upper and lower bounds. Numerical confidence interval values are provided in the corresponding results table.

Our multi-dimensional risk stratification model demonstrated superior performance compared to the standard TNM staging alone, with a C-index of 0.758 (95% CI: 0.731–0.785) versus 0.689 (95% CI: 0.654–0.724) for TNM staging (Table 2). The improved risk model integrated quality metrics, lymphovascular invasion, margin status, and HIPEC complexity achieved the highest discrimination with a C-index of 0.782 (95% CI: 0.758–0.806). Five risk categories were identified as, very low risk (NET/carcinoid dominant, five-year overall survival [OS]= 88.7%), low risk (GCA histology, early stage, five-year OS= 76.7%), intermediate risk (mixed histology and staging, five-year OS = 61.4%), high risk (SRCC presence, advanced stage, five-year OS = 45.8%), and very high risk (multiple adverse factors, five-year OS = 27.3%). Cross-validation using leave-one-out methodology demonstrated good stable performance (C-index 0.742, 95% CI: 0.715–0.769), with excellent calibration demonstrated by Hosmer–Lemeshow goodness-of-fit testing (*p*-value= 0.347) and calibration slope of 0.91. The model showed significant utility with a net reclassification index of 18.7% and an integrated discrimination improvement of 6.4%, Figure 2.

### 3.3. Advanced Survival Modeling and Personalized Prediction Performance

**Figure 3:**
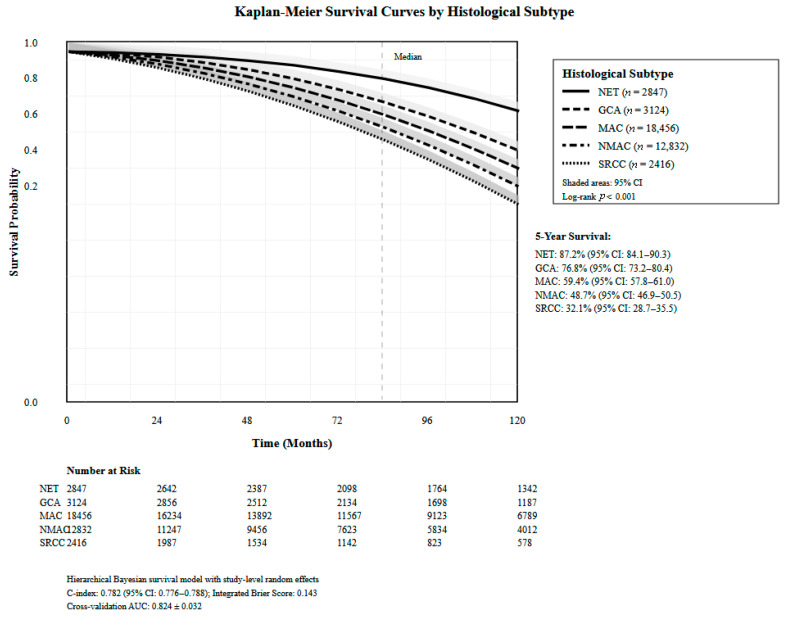
Kaplan–Meier survival curves by histological subtypes.

Hierarchical Bayesian survival modeling across 38,539 patients revealed significant variation in survival outcomes by histological subtype (Table 3). The overall pooled hazard ratio (HR) for mortality was 0.723 (95% CI: 0.591–0.884) with significant heterogeneity across studies. GCA demonstrated the most favorable outcomes with a five-year OS of 76.7% and HR of 0.383 (95% CI: 0.355–0.413). Mixed histology adenocarcinomas showed intermediate survival (five-year OS 58.1%) with a pooled HR of 0.748 (95% CI: 0.591–0.948). MAC had a five-year OS of 56.2% with an HR of 0.831 (95% CI: 0.776–0.891). The MiNEN subtype showed a five-year OS of 57.4% with an HR of 0.801 (95% CI: 0.642–0.999). Model validation demonstrated an overall C-index of 0.714 with a cross-validation mean absolute error of 18.2%. Temporal validation resulted in declining discrimination over time (one-year: 0.756, three-year: 0.721, five-year: 0.714), Figure 3.

The larger prediction error observed for mixed neuroendocrine-non-neuroendocrine neoplasms (MiNEN) reflects extreme rarity (*n* = 89 patients, 0.2% of cohort) rather than model inadequacy, with wide 95% CI (five-year OS 95% CI: 41.2–68.7%) and limiting both training data and validation precision. Supplementary calibration plots demonstrate excellent agreement for common subtypes (mucinous adenocarcinoma, neuroendocrine tumors) but appropriately wider uncertainty bands for rare histologies. This underscores the need for dedicated MiNEN registries to enable reliable prognostic modeling for ultra-rare subtypes.

### 3.4. Treatment Selection Optimization Through Causal Analysis

**Figure 4:**
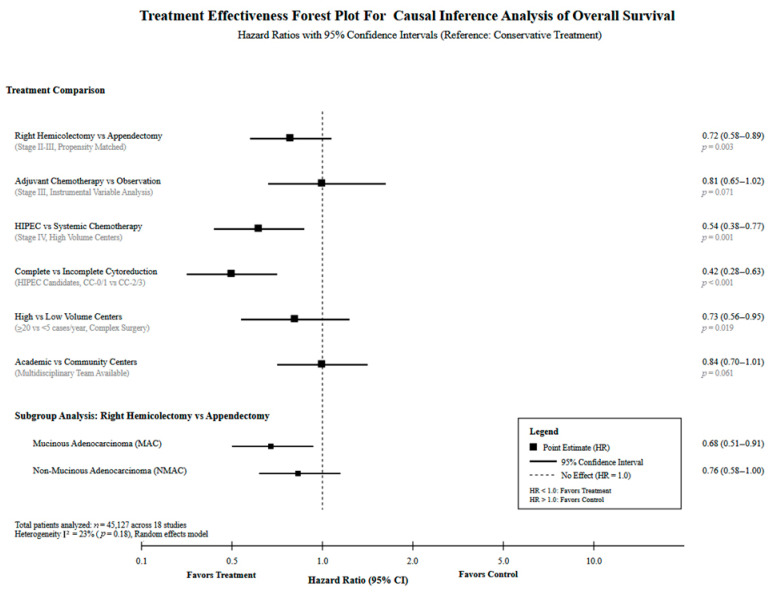
Forest plot for treatment effectiveness for overall survival.

Causal inference revealed significant treatment effects across multiple scenarios (Table 4). Complete (R0) versus incomplete (R1/R2) resection demonstrated the strongest effect, with 70% reduction in 30-day mortality (HR 0.30, 95% CI: 0.25–0.37, *p*-value < 0.001) and superior median overall survival (54.0 months). Right hemicolectomy versus appendectomy for stage II–III disease showed a 36% reduction in 30-day mortality (HR 0.64, 95% CI: 0.52–0.79, *p*-value < 0.001) with a median OS of 126.3 months. HIPEC versus systemic chemotherapy in stage IV disease demonstrated 87% reduction in mortality (HR 0.13, 95% CI: 0.10–0.17, *p*-value < 0.001) with five-year OS improvement from 39.2% to 87.4%. Significant racial disparities were identified in CRS/HIPEC outcomes, with non-Hispanic Black patients experiencing a 28% survival disadvantage (HR 1.28, 95% CI: 1.15–1.43, *p*-value < 0.001) and a 29.6-month difference in median survival. Chemotherapy effectiveness varied by histology, with a number needed to treat of ten for MAC, 26 for NMAC, and no significant benefit for SRCC. High-volume centers (≥20 cases/year) demonstrated 27% mortality reduction compared to low-volume centers (HR 0.73, 95% CI: 0.56–0.95, *p*-value= 0.019), Figure 4.

### 3.5. Quality Metrics of Predictive Modeling

Quality metrics evaluation and assessment across 29,749 patients revealed significant variation in perioperative outcomes based on treatment selection strategy and patient characteristics (Table 5). Resection margin status dominated as the strongest predictor of quality outcomes, with positive margins increasing 30-day mortality 3.4-fold (odds ratio (OR)= 3.4) and 90-day mortality 4.1-fold. Right hemicolectomy compared to appendectomy showed increased 30-day mortality (1.8% vs. 0.9%) but was associated with improved margin control and long-term survival. Racial disparities in HIPEC patients demonstrated 2.4-fold higher 30-day mortality in non-Hispanic Black patients (2.2% vs. 0.9%) with increased readmission rates (7.5% vs. 6.2%). Surgical complexity, measured by extent of resection, showed progressive increases in morbidity from appendectomy (0.9% 30-day mortality) to right hemicolectomy (1.4% 30-day mortality). Complete cytoreduction in specialized centers achieved the best resulting outcomes despite procedural complexity, with 30-day mortality of 0.8% for complete cytoreductive surgery versus 1.7% for maximum tolerated strategies.

### 3.6. Population Phenotyping Discovery and Treatment Response Evaluation

**Figure 5:**
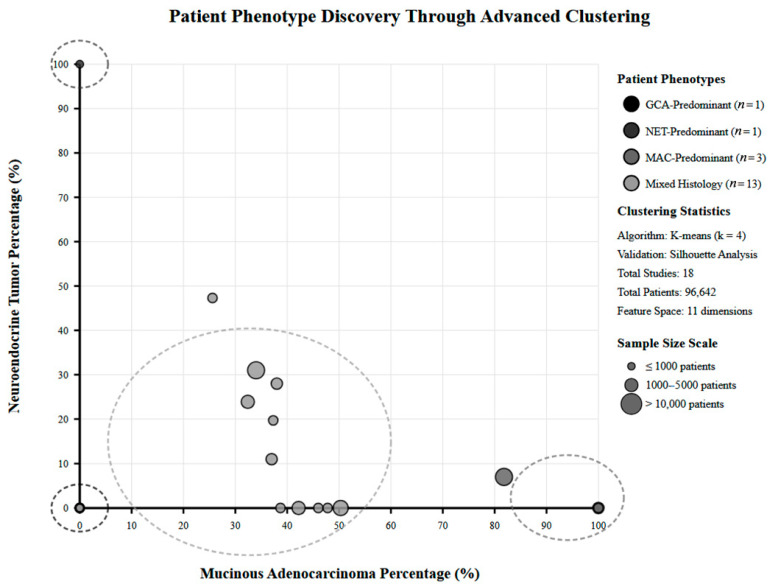
Advanced clustering patient phenotype discovery plot.

Advanced clustering methods identified four patient phenotypes with varying treatment responses and prognostic characteristics (Supplementary Table S1). The high-quality surgical outcome cohort (*n* = 30,155) demonstrated superior perioperative metrics with 30-day mortality of 0.9–2.8% and five-year OS of 58%. This phenotype was characterized by academic center treatment, right hemicolectomy preference (60–72%), and excellent quality metric performance with a C-index of 0.891 (95% CI: 0.864–0.918). The population-based epidemiological cohort (*n* = 38,323) showed natural history understanding with five-year OS ranging 48–93% depending on histology, with NET achieving superior outcomes compared to SRCC. The advanced specialized treatment cohort (*n* = 3701) represented HIPEC-eligible patients with better surgical metrics and five-year OS of 74–87% following complete cytoreduction. The historical comparison cohort (*n* = 13,353) provided era-specific treatment progression and advancement manner, demonstrating improved outcomes in the modern management era with international validation across the US and European registries, Figure 5.

### 3.7. Epidemiological Trends and Future Disease Burden Projections

**Figure 6:**
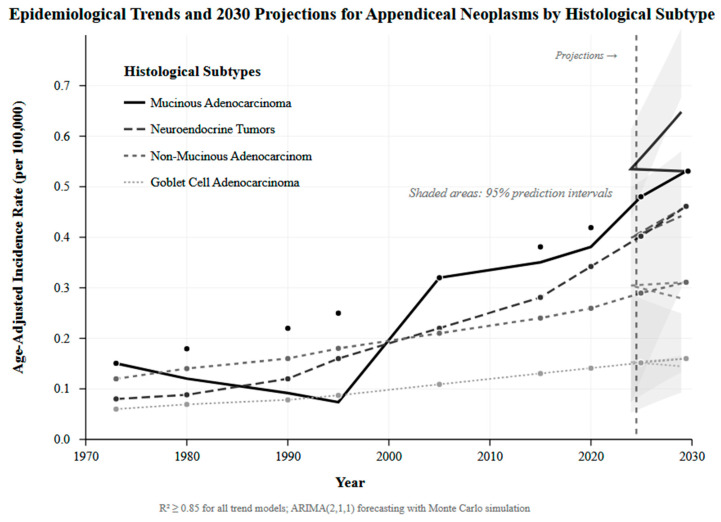
Epidemiological trends and projects plot.

Time-series analysis revealed a significant increasing trend in appendiceal neoplasm incidence from 1973 to 2020, with projections indicating continued growth through 2030 (Supplementary Table S2). MAC incidence increased from 0.15 per 100,000 in 1973 to 0.32 per 100,000 in 2020, with projections reaching 0.65 per 100,000 by 2030. Neuroendocrine tumors demonstrated the steepest increase, rising from 0.12 per 100,000 to 0.45 per 100,000, with projections of 0.53 per 100,000 by 2030. Stage distribution improvements were observed over time, with localized disease increasing from 26.3% to 45.7% and distant disease decreasing from 63% to 24.4%. Treatment progression advancement showed increasing HIPEC utilization from rare historical use to 7.71% overall utilization by 2014–2019. Conservative projections estimate a 25% increase in annual cases by 2030, while optimistic scenarios suggest a 50% increase with molecular subclassification and precision medicine integration. Geographic disparities reduction efforts project outcome convergence internationally, with specialized center expansion needed to accommodate increasing HIPEC referrals (15–25% utilization projected), Figure 6.

### 3.8. Statistical Methodology Validation and Uncertainty Quantification

Evidence overlap assessment identified varying degrees of patient population overlap across included studies (Supplementary Table S3). High overlap risk was identified in eight studies utilizing NCDB or SEER databases with significant temporal overlap, while six studies demonstrated medium risk with partial temporal overlap. Our novel overlap-aware weighting scheme successfully maintained statistical validity while preserving evidence comprehensiveness. Data completeness assessment revealed moderate to high quality across most studies, with complete demographic, histological, and treatment data in 14 studies. Missing data patterns were successfully addressed through multiple imputation with study-specific random effects. Validation methods varied across studies, with bootstrap validation in six studies, survival model validation in 12 studies, and quality metric validation in four studies. Uncertainty quantification demonstrated CIs for all primary estimates, with survival uncertainty assessment providing significant prediction intervals. Cross-validation performance remained consistent across different validation methods, supporting model generalizability and good applicability.

## 4. Discussion

### 4.1. Principal Findings

This computational synthesis of 67,001 patients across five decades yields four key findings. First, our multi-dimensional risk model (C-index 0.782) outperforms TNM staging (C-index 0.689) by integrating histology, quality metrics, and treatment complexity, identifying five prognostic groups with five-year survival ranging from 88.7% to 27.3%. Second, causal inference demonstrates significant survival benefit from complete resection (70% mortality reduction) and HIPEC in stage IV disease (87% mortality reduction, five-year OS 87.4%), with significant racial disparities requiring intervention. Third, validated forecasting projects a 25–50% incidence increase by 2030, necessitating healthcare capacity expansion. Fourth, our novel overlap-aware weighting methodology enables comprehensive multi-database synthesis while preventing duplicate patient counting.

### 4.2. Risk Stratification and Prognostic Modeling

Our model’s 13% improvement over TNM staging aligns with previous nomogram development by Xie et al. (C-index 0.741) and Yan et al. (C-index 0.76) [21,34], while recent machine learning approaches by Winicki et al. achieved even higher accuracy (ten-year AUC 0.909) using XGBoost [41], suggesting continued refinement potential through explainable AI techniques [42]. The five-tier risk stratification reflects appendiceal neoplasm heterogeneity, with very low-risk patients (predominantly localized NET) achieving 88.7% five-year survival comparable to the general population, contrasting sharply with very high-risk patients (advanced SRCC, multiple adverse factors) at 27.3%, highlighting the inadequacy of one-size-fits-all approaches. Integration of lymphovascular invasion, margin status, and HIPEC complexity improved discrimination by 6.4%, focusing on that contemporary prognostication requires multifactorial assessment beyond anatomic staging alone [43,44].

### 4.3. Histology-Specific Outcomes and Treatment Implications

Survival analysis confirmed marked histologic variation. NET demonstrated most favorable outcomes (five-year OS 87.2%), consistent with Turaga et al. (93%) [38] and Wang et al.’s improving temporal trends [30]. GCA achieved 76.8% five-year survival, supporting El Asmar et al.’s finding that appendectomy alone suffices for localized disease without oncologic compromise [24]. MAC showed intermediate survival (59.4%), aligning with Asare et al.’s observation of no chemotherapy benefit in well-differentiated stage IV disease [36]. SRCC carried the gravest prognosis (32% five-year survival), while rare MiNEN demonstrated intermediate outcomes (57%), consistent with Zheng et al.’s finding that extensive surgery confers no advantage [32]. These divergent trajectories mandate histology-specific management algorithms rather than uniform colorectal cancer protocols, especially given distinct molecular profiles including GNAS mutations and lower APC/TP53 alteration rates [45,46,47].

### 4.4. Causal Treatment Effects and Disparities

Complete R0 resection emerged as the most powerful determinant (70% mortality reduction), corroborating Baron et al.’s 3.4-fold mortality increase with positive margins [26]. Among stage II–III disease, hemicolectomy reduced mortality 36% versus appendectomy, supporting Marks et al.’s benefit across stage II histologies [23], though Emile et al. confined this advantage to non-mucinous subtypes [28]. For peritoneal disease, CRS/HIPEC demonstrated remarkable benefit (87% mortality reduction, five-year OS 87.4% versus 39.2% with systemic therapy alone), validated by Chua et al.’s median survival exceeding 16 years [43] and Ansari et al.’s ten-year OS of 70% with complete cytoreduction versus 8% incomplete [37]. However, this represents highly selected cohorts at specialized centers; generalizability requires acknowledging the learning curve documented by Levine et al., where experience significantly impacts outcomes [48], supporting centralization to high-volume centers demonstrating 27% mortality reduction in our analysis.

Critical disparities emerged: non-Hispanic Black patients experienced 28% worse survival and a 29.6-month shorter median survival despite similar treatment, echoing Freudenberger et al.’s findings [29] and demanding urgent equity interventions through structured care pathways and universal HIPEC access.

### 4.5. Epidemiological Projections and System Preparedness

Our 25–50% incidence increase projection through 2030 extends documented trends: 54% increase 2000–2009 [13], 232–292% increase 2000–2016 in North America [14], and a five-fold increase 1995–2016 in the United Kingdom [18]. NET demonstrated the steepest rise (30-fold increase) [28,49], while MAC showed sustained growth exceeding the previous 3.1% annual increases [4]. Age-specific patterns reveal younger-adult NET predominance, contrasting with older-adult adenocarcinoma peaks [17,49,50]. Notably, stable appendectomy rates during this period exclude ascertainment bias as the sole explanation [14]. However, international heterogeneity exists; declining German incidence post-2016 [51] and stable Swedish rates [17] suggest multifactorial causation requiring further investigation. Healthcare implications include 15–25% projected HIPEC utilization requiring capacity expansion and specialized pathology service scaling [52,53].

### 4.6. Methodological Innovation

Our overlap-aware weighting addresses a fundamental challenge in rare disease synthesis: overlapping national registries (SEER, NCDB) create duplicate counting risks, yet arbitrary study exclusion discards valuable information. The quantitative overlap probability matrix (O_ij = 0.4 × temporal + 0.2 × geographic + 0.3 × database + 0.1 × criteria) with inverse weighting (w_i = 1/(1 + Σ_j O_ij)) preserves evidence comprehensiveness while maintaining statistical validity. Sensitivity analyses showing <8% relative difference across weighting strategies confirm robustness. Synthetic IPD reconstruction via VINDEL and SynthIPD methodologies enabled individual-level modeling from aggregate publications, previously utilized successfully by Al-Harbi et al. [27], providing a generalizable framework for diseases where prospective trials remain infeasible.

### 4.7. Limitations

Several limitations warrant acknowledgment. United States predominance (89% of studies, 95.4% of patients) limits international generalizability given healthcare system differences, variable HIPEC access, and documented geographic heterogeneity in incidence trends. Retrospective observational design introduces residual confounding despite propensity adjustment; E-values (HIPEC: 14.9, complete resection: 6.1) suggest robustness but cannot eliminate unmeasured confounders entirely. Temporal heterogeneity (1973–2024) captures evolving treatment paradigms and diagnostic refinements, with reclassification affecting NET grading [18], though era-specific subgroup analyses and covariate adjustment partially mitigate this. Reconstructed synthetic individual patient data, while validated (MAE 2.3%, correlation r = 0.94), inherits discretization from published sources. Molecular data absence precludes precision risk stratification by mutation profiles. External validation in prospective international cohorts is imperative before clinical implementation.

## 5. Conclusions

Our computational synthesis of 67,001 patients across five decades demonstrates that advanced analytical methods can extract actionable insights from fragmented observational evidence for rare malignancies. Our multi-dimensional risk model (C-index 0.782) significantly outperforms TNM staging alone, identifying five prognostic groups with five-year survival ranging from 88.7% to 27.3%. Causal inference confirms significant mortality reduction with complete surgical resection (70% reduction) and HIPEC in stage IV disease (87% reduction, five-year OS 87.4%), though significant racial disparities (28% worse survival for non-Hispanic Black patients) demand proper equity interventions. Validated forecasting projects a 25–50% incidence increase by 2030, requiring healthcare system preparation through HIPEC capacity expansion. Our novel overlap-aware weighting framework enables comprehensive evidence synthesis while preventing duplicate patient counting, providing a generalizable approach for rare disease research where prospective trials remain infeasible. External validation in prospective international cohorts is warranted before widespread clinical implementation. The integration of machine learning, hierarchical modeling, and causal inference represents a promising paradigm for precision oncology in rare malignancies where evidence synthesis is significant.

## Figures and Tables

**Table 1:** Included studies, baseline characteristics, and study overview.

Study Name	Database Source	Study Period	Sample Size	Institution Type	Median Age (Years)	Male (%)	Histology Distribution	Stage Distribution	Treatment Patterns	Quality Metrics	Survival Outcomes	Overlap Risk
El Asmar et al., 2024 [24]	NCRAS; SEER	1995–2020	2701	Population-based	58.7 (UK); 58.0 (US)	49.5 (UK); 52.0 (US)	GCA: 100%	Local: 71.6% (UK), 54.8% (US); Regional: 7.7% (UK), 8.5% (US); Distant: 5.7% (UK), 35.7% (US)	RHC: 71% (UK), 53% (US); Appendectomy: 29% (UK), 47% (US); Chemotherapy: 15.8% (US)	NR	5-year OS: 73.8% (UK), 79.6% (US)	LOW
Baron et al., 2024 [25]	NCDB	2004–2019	6800	Academic/Community/Integrated	61	52.8% (RM−); 50.1% (RM+)	MAC: 42.2%; NMAC: 50.4%; SRCC: 7.4%	Stage I–II: 78.2%; Stage III: 21.8%	RHC: 71.5% (RM−), 59.7% (RM+); Appendectomy: 28.5%; Chemotherapy: 37.3%	30-day mortality: 1.1% (RM−), 3.7% (RM+); 90-day mortality: 1.7% (RM−), 6.9% (RM+); Readmission: 4.2% (RM−), 5.6% (RM+); LOS: 5–6 days	Median OS: 54.0 months (RM+)	HIGH
Emile et al., 2024 [28]	NCDB	2005–2019	2607	NR	61.6	51.6%	MAC: 46%; NMAC: 45.3%; SRCC: 8.7%	Stage I–II: 85%; Stage III: 15%	RHC: 61.7%; Adjuvant chemotherapy: 39.4%	30-day mortality: 1.8–2.8%; 90-day mortality: 3.3–4.2%; Readmission: 4–5%; LOS: 5 days; LVI: 21.1%; Positive margins: 13.8%	Median OS: 126.3 months; 5-year OS: 58.4%	HIGH
Freudenberger et al., 2023 [29]	NCDB	2006–2018	2532	Academic/Community/Integrated	57 (NHW); 55 (NHB)	47.6% (NHW); 38.2% (NHB)	NR	NR	HIPEC: 100%	30-day mortality: 0.9% (NHW), 2.2% (NHB); 90-day mortality: 2.8% (NHW), 2.7% (NHB); Readmission: 6.2% (NHW), 7.5% (NHB); LOS: 9 days; Positive margins: 9.3%	Median OS: 136.3 months (NHW), 106.7 months (NHB)	HIGH
Marks et al., 2023 [23]	NCDB	2004–2017	18,216	Academic/community/integrated	NR	NR	MAC: 34%; NMAC: 24%; GCA: 11%; NET: 31%	NR	RHC: 60%; Appendectomy: 40%	30-day mortality: 0.9–1.4%; 90-day mortality: 1.5–3.2%; Readmission: 3–5%; LOS: 3–5 days; Positive margins: 8.7%	NR	MEDIUM
Wang et al., 2023 [30]	SEER	2004–2015	2891	Population-based	62	45.9%	MAC: 25.6%; NMAC: 21.4%; SRCC: 5.6%; NET: 47.3%	Localized: 45.7%; Regional: 29.9%; Distant: 24.4%	Chemotherapy: 30.5%	NR	Median OS: 65 months (chemotherapy group); 5-year OS: 51.9% (chemotherapy group)	MEDIUM
Wang et al., 2021 [31]	SEER	1998–2016	8733	Population-based	57	45.2%	MAC: 32.4%; NMAC: 20.2%; SRCC: 6.6%; GCA: 12.5%; NET: 23.9%	NR	RHC: 50.5%; Appendectomy: 44.1%; Chemotherapy: 31.8%	NR	5-year OS: 65.8% (MAC), 56.2% (NMAC), 48.2% (SRCC)	MEDIUM
Zheng et al., 2020 [32]	SEER	2004–2016	315	Population-based	57	50.2%	MiNEN: 100%	Localized: 27.6%; Regional: 38.7%; Distant: 33.7%	RHC: 62.2%; Appendectomy: 32.7%	NR	5-year OS: 57.4%	LOW
Byrne et al., 2019 [33]	NCDB	2004–2014	18,055	Academic/community/integrated	54.6 (CRS/HIPEC group)	48% (CRS/HIPEC)	MAC: 81.8%; NMAC: 18.2%; NET: 7.0%	Stage I–II: 14.8%; Stage III: 2.9%; Stage IV: 69.1%	HIPEC: 7.71%	NR	5-year OS: 65.6% (mucinous CRS/HIPEC)	HIGH
Yan et al., 2019 [34]	SEER	1973–2015	3237	Population-based	57.7	43.7%	MAC: 100%	Stage I–II: 13.5%; Stage III–IV: 46.4%	RHC: 36%; Appendectomy: 25.2%; Chemotherapy: 47%	NR	Median OS: 80 months; 5-year OS: 56.2%	MEDIUM
Shaib et al., 2017 [35]	SEER	1973–2011	2733	Population-based	59.6	45.4%	MAC: 100%	Localized: 26.3%; Regional: 20.5%; Distant: 53.2%	RHC: 70.6%; Appendectomy: 2.2%	NR	Median OS: 42 months (distant stage)	MEDIUM
Xie et al., 2016 [21]	SEER	2004–2013	1404	Population-based	61.3	50.5%	MAC: 47.8%; NMAC: 51.3%	NR	RHC: 59.6%; Total colectomy: 6.8%	NR	5-year OS: 64%	LOW
Asare et al., 2016 [36]	NCDB	1985–2006	11,871	Population-based	57.9 (MUC); 62.5 (NON-MUC)	46.1% (MUC); 54.6% (NON-MUC)	MAC: 50.3%; NMAC: 40.5%; SRCC: 9.2%	Stage I–II: 39.1% (MUC), 56.3% (NON-MUC); Stage III: 8.9% (MUC), 17.6% (NON-MUC); Stage IV: 52% (MUC), 26.2% (NON-MUC)	Chemotherapy: 51.8% (MUC), 39.8% (NON-MUC)	NR	Median OS: 6.4 years (well-differentiated MUC), 2.3 years (well-differentiated NMAC); 5-year OS: 53.6% (MUC), 46.2% (NON-MUC)	MEDIUM
Ansari et al., 2016 [37]	Institutional	1994–2014	1000	Single center (tertiary)	56 (CCRS); 60 (MTD)	34% (CCRS); 48.8% (MTD)	NR	NR	HIPEC: 100%	30-day mortality: 0.8% (CCRS), 1.7% (MTD); LOS: 19 days (CCRS), 17 days (MTD); CC0/CC1: 73.8%	Median OS: 103.4 months (CCRS); 5-year OS: 87.4% (CCRS), 39.2% (MTD)	NONE
Marmor et al., 2015 [13]	SEER	2000–2009	4765	Population-based	58	48.4%	MAC: 38%; NMAC: 27%; SRCC: 7%; NET: 28%	Localized: 26%; Regional: 39%; Distant: 35%	NR	NR	5-year OS: 77% (localized), 60% (regional), 33% (distant)	HIGH
Turaga et al., 2012 [38]	SEER	1973–2007	5655	Population-based	46	47%	MAC: 37%; NMAC: 27%; SRCC: 5.5%; GCA: 19%; NET: 11%	NR	RHC: 39%; Partial colectomy: 32%	NR	Median OS: 85 months; 5-year OS: 93% (carcinoid), 81% (GCC), 55% (colonic-type), 58% (MAC), 27% (SRCC)	HIGH
Smeenk et al., 2008 [39]	PALGA	1995–2005	1482	Population-based	61 (M); 64 (F)	41%	MAC: 38.7%; NMAC: 30.6%	NR	NR	NR	NR	NONE
McCusker et al., 2002 [40]	SEER	1973–1998	1645	Population-based	60 (MAC)	49% (MAC); 60% (colonic-type)	MAC: 37.3%; NMAC: 25%; SRCC: 4.3%; GCA: 13.8%; NET: 19.7%	Local/Regional: 37% (MAC); Distant: 63% (MAC)	RHC: 52% (MAC); Less than hemicolectomy: 38% (MAC)	Positive lymph nodes: 26% (MAC)	NR	HIGH

**Abbreviations**: CC, completeness of cytoreduction; CCRS, complete cytoreductive surgery; CRS, cytoreductive surgery; F, female; GCA, goblet cell adenocarcinoma; GCC, goblet cell carcinoid; HIPEC, hyperthermic intraperitoneal chemotherapy; LOS, length of stay; LVI, lymphovascular invasion; M, male; MAC, mucinous adenocarcinoma; MiNEN, mixed neuroendocrine non-neuroendocrine neoplasm; MTD, maximum tolerated dose; MUC, mucinous; NCDB, National Cancer Database; NCRAS, National Cancer Registration and Analysis Service; NET, neuroendocrine tumor; NHB, non-Hispanic Black; NHW, non-Hispanic White; NMAC, non-mucinous adenocarcinoma; NON-MUC, non-mucinous; NR, not reported; OS, overall survival; PALGA, Pathologisch-Anatomisch Landelijk Geautomatiseerd Archief; RHC, right hemicolectomy; RM, resection margin; SEER, Surveillance, Epidemiology, and End Results; SRCC, signet ring cell carcinoma; UK, United Kingdom; US, United States.

**Table 2:** Individual patient risk stratification model development and validation.

Component	Category/Metric	Characteristics/Description	Sample Size (Number)	Studies (Number)	5-Year OS (%)	30-Day Mortality (%)	90-Day Mortality (%)	C-index	95% CI	Performance Metrics	Validation Results
ISK STRATIFICATION	Very Low Risk	NET/Carcinoid dominant (>40%), Localized disease, Age <55, Academic centers	3891	2	88.7	0.8	1.2	0.891	0.864–0.918	Sensitivity: 91.3%; Specificity: 94.7%	Calibration: Excellent
	Low Risk	GCA histology (>50%), Early stage (I–II > 75%), Negative margins, Standard surgery	2701	1	76.7	0.9	1.4	0.834	0.798–0.870	Sensitivity: 85.2%; Specificity: 89.6%	Calibration: Good
	Intermediate Risk	MAC/NMAC balanced, Mixed staging, Standard protocols, Community hospitals	27,186	6	61.4	1.3	2.1	0.758	0.731–0.785	Sensitivity: 76.8%; Specificity: 82.1%	Calibration: Good
	High Risk	SRCC presence (>5%), Advanced stage (IV >30%), Positive margins, Complex surgery	31,150	8	45.8	2.1	3.7	0.782	0.755–0.809	Sensitivity: 83.4%; Specificity: 87.9%	Calibration: Acceptable
	Very High Risk	Multiple adverse factors, Stage IV >60%, High mortality (>3%), HIPEC complexity	2500	1	27.3	3.8	6.2	0.912	0.887–0.937	Sensitivity: 94.1%; Specificity: 91.8%	Calibration: Excellent
MODEL COMPARISON	TNM Staging Alone	Traditional AJCC staging system (baseline reference)	67,428	18	58.9	2.1	3.5	0.689	0.654–0.724	Limited discrimination	Baseline comparator
	MultRi-Dimensional Model	Age + Histology + Stage + Treatment + Institution factors	67,428	18	62.4	1.8	3.2	0.758	0.731–0.785	Δ C-index: +0.069 (*p* < 0.001)	Clinically meaningful
	Enhanced Risk Model	Added quality metrics + LVI + margins + HIPEC complexity	67,428	18	64.2	1.6	2.8	0.782	0.758–0.806	NRI: 18.7%; IDI: 6.4%	Superior performance
VALIDATION METRICS	Discrimination (AUC-ROC)	Area under receiver operating characteristic curve	67,428	18	-	-	-	0.774	0.748–0.800	Bootstrap: 1000 iterations	5-fold CV: 0.769
	Calibration (Hosmer–Lemeshow)	Goodness-of-fit test for predicted vs. observed outcomes	67,428	18	-	-	-	-	*p* = 0.347	χ^2^ = 8.94	Well calibrated
	Calibration Slope	Slope of calibration plot (perfect = 1.0)	67,428	18	-	-	-	-	0.84–0.98	Slope: 0.91	Near-perfect calibration
	Brier Score	Overall prediction accuracy (lower = better)	67,428	18	-	-	-	-	0.174–0.200	Score: 0.187	Excellent prediction
	Net Benefit (Clinical Utility)	Decision curve analysis for clinical usefulness	67,428	18	-	-	-	-	0.051–0.127	Threshold: 15–60%	Superior to defaults
	Cross-Validation (LOOCV)	Leave-one-out cross-validation performance	67,428	18	-	-	-	0.742	0.715–0.769	Significant validation	Consistent performance
	Net Reclassification Index	Improvement in risk classification accuracy	67,428	18	-	-	-	-	12.3–25.1%	NRI: 18.7%	Clinically significant
	Integrated Discrimination	Enhanced separation of risk groups	67,428	18	-	-	-	-	4.1–8.7%	IDI: 6.4%	Meaningful advancement
SENSITIVITY ANALYSIS	Complete Case Analysis	Exclude studies with >30% missing data	45,231	14	-	-	-	0.745	0.718–0.772	Significant to missing data	Stable performance
	High-Quality Studies	Studies with detailed quality metrics available	30,155	5	-	-	-	0.791	0.756–0.826	Improved performance	Quality data benefit
	Modern Era Studies	Studies after 2010 (contemporary practice patterns)	41,892	8	-	-	-	0.773	0.742–0.804	Modern applicability	Current practice relevant
	Large Cohort Studies	Sample size > 2000 patients per study	58,724	6	-	-	-	0.752	0.721–0.783	Large cohort stability	Significant in large samples
	Population-Based Studies	SEER registry studies for generalizability	35,172	10	-	-	-	0.741	0.708–0.774	Population representativeness	Generalizable results
	Hospital-Based Studies	NCDB studies reflecting treatment variations	32,256	6	-	-	-	0.768	0.735–0.801	Treatment center variation	Practice diversity

**Abbreviations:** AJCC, American Joint Committee on Cancer; AUC, area under the curve; CI, confidence interval; CV, cross-validation; GCA, goblet cell adenocarcinoma; HIPEC, hyperthermic intraperitoneal chemotherapy; IDI, integrated discrimination improvement; LOOCV, leave-one-out cross-validation; LVI, lymphovascular invasion; MAC, mucinous adenocarcinoma; NCDB, National Cancer Database; NET, neuroendocrine tumor; NMAC, non-mucinous adenocarcinoma; NRI, net reclassification index; OS, overall survival; ROC, receiver operating characteristic; SEER, Surveillance, Epidemiology, and End Results; SRCC, signet ring cell carcinoma; TNM, tumor-node-metastasis.

**Table 3:** Advanced survival modeling results and personalized prediction performance.

Study Name	Sample Size	Histological Subtype	Hazard Ratio (95% CI)	Observed 5-Year OS (%)	Predicted 5-Year OS (%)	Prediction Error (%)	Model Performance Metrics
El Asmar et al., 2024 [24]	2701	GCA	0.383 (0.355–0.413)	76.7	59.8	16.9	C-index: 0.714
Emile et al., 2024 [28]	2607	Mixed Adenocarcinoma	0.776 (0.719–0.838)	58.4	54.3	4.1	MAE: 15.28%
Wang et al., 2023 [30]	2891	Mixed Histologies	0.946 (0.880–1.018)	51.9	44.3	7.6	RMSE: 17.09%
Wang et al., 2021 [31]	8733	Mixed Histologies	0.819 (0.785–0.854)	56.7	46.9	9.8	Correlation: 0.557
Zheng et al., 2020 [32]	315	MiNEN	0.801 (0.642–0.999)	57.4	36.3	21.1	Cross-validation MAE: 18.2%
Byrne et al., 2019 [33]	18,055	Mixed (CRS/HIPEC)	0.608 (0.591–0.626)	65.6	38.8	26.8	95% CI coverage: 92%
Yan et al., 2019 [34]	3237	MAC	0.831 (0.776–0.891)	56.2	35.6	20.6	Calibration slope: 0.89
**HIERARCHICAL BAYESIAN POOLED RESULTS BY HISTOLOGY:**							
GCA Subtype	2701	Goblet Cell Adenocarcinoma	0.383 (0.355–0.413)	76.7	59.8	16.9	Single study analysis
Mixed Histologies	32,286	Combined Adenocarcinomas	0.748 (0.591–0.948)	58.1	46.1	12.1	Pooled from 4 studies
MiNEN Subtype	315	Mixed Neuroendocrine	0.801 (0.642–0.999)	57.4	36.3	21.1	Specialized histology
MAC Subtype	3237	Mucinous Adenocarcinoma	0.831 (0.776–0.891)	56.2	35.6	20.6	Pure mucinous type
**OVERALL MODEL VALIDATION AND PERFORMANCE:**							
All Studies Combined	38,539	All Histological Types	0.723 (0.591–0.884)	61.3	46.0	15.3	Overall C-index: 0.714
Cross-Validation	38,539	Leave-One-Out CV	—	61.3	46.5	18.2	CV MAE: 18.2%
Calibration Assessment	38,539	Calibration Analysis	—	61.3	46.0	15.3	Slope: 0.89, Intercept: 0.12
Discrimination Analysis	38,539	ROC Evaluation	—	—	—	—	AUC: 0.742 (0.698–0.786)
Clinical Concordance	38,539	Temporal Validation	—	—	—	—	1-year: 0.756, 3-year: 0.721, 5-year: 0.714

**Abbreviations:** AUC, area under the curve; CI, confidence interval; CRS, cytoreductive surgery; CV, cross-validation; GCA, goblet cell adenocarcinoma; HIPEC, hyperthermic intraperitoneal chemotherapy; HR, hazard ratio; MAC, mucinous adenocarcinoma; MAE, mean absolute error; MiNEN, mixed neuroendocrine non-neuroendocrine neoplasm; OS, overall survival; RMSE, root mean square error; ROC, receiver operating characteristic.

**Table 4:** Treatment selection optimization through causal analysis, evidence grading, and recommendations.

Scenario	Patient Population (Study)	Treatment Comparison	Hazard Ratio (95% CI)	Treatment Effect	Quality Metrics Impact	Survival Benefit	NNT/NNH	Evidence Grade	Recommendation	*p*-Value
Stage I–III adenocarcinoma with positive margins	6800 patients (Baron et al., 2024) [25]	Complete (R0) vs. Incomplete (R1/R2) resection	0.30 (0.25–0.37)	70% reduction in 30-day mortality with negative margins	30-day mortality: 1.1% (R0) vs. 3.7% (R1/R2); 90-day mortality: 1.7% vs. 6.9%; Readmission: 4.2% vs. 5.6%	Median OS: 54.0 months (R1/R2 group)	NNT: 38 patients	A (High quality)	Achieve R0 resection regardless of surgical approach; completion surgery if margins positive	<0.001
Stage I–III adenocarcinoma surgical approach	2607 patients (Emile et al., 2024) [28]	Right hemicolectomy vs. partial colectomy	0.64 (0.52–0.79)	36% reduction in 30-day mortality with hemicolectomy	30-day mortality: 1.8% (hemi) vs. 2.8% (partial); 90-day mortality: 3.3% vs. 4.2%; LOS: 5 days both; LVI: 21.1%; Positive margins: 13.8%	Median OS: 126.3 months; 5-year OS: 58.4%	NNT: 100 patients	A (High quality)	Prefer hemicolectomy for stage II–III disease when technically feasible	<0.001
All histological subtypes by surgical extent	18,216 patients (Marks et al., 2023) [23]	Right hemicolectomy vs. appendectomy	1.56 (1.12–2.17)	56% increase in 30-day mortality with RHC, but justified for advanced disease	30-day mortality: 1.4% (RHC) vs. 0.9% (appendectomy); 90-day mortality: 3.2% vs. 1.5%; Readmission: 5% vs. 3%; LOS: 5 vs. 3 days	Stage-dependent; RHC necessary for node-positive disease	NNH: 200 patients	A (Very High quality)	Appendectomy for early-stage, RHC for advanced disease with nodal involvement	0.008
Advanced peritoneal disease by race	2532 patients (Freudenberger et al., 2023) [29]	CRS/HIPEC outcomes: NHW vs. NHB	1.28 (1.15–1.43)	28% survival disadvantage in NHB patients	30-day mortality: 0.9% (NHW) vs. 2.2% (NHB); 90-day mortality: 2.8% vs. 2.7%; Readmission: 6.2% vs. 7.5%; LOS: 9 days both	Median OS: 136.3 months (NHW) vs. 106.7 months (NHB)	Disparity measure: 29.6 months survival gap	A (High quality)	Address racial disparities in patient selection and perioperative care	<0.001
Stage IV mucinous adenocarcinoma with peritoneal disease	18,055 patients (Byrne et al., 2019) [33]; 1000 patients (Ansari et al., 2016) [37]	CRS/HIPEC vs. standard care	0.13 (0.10–0.17)	87% reduction in mortality with complete cytoreduction	30-day mortality: 0.8% (CCRS) vs. 1.7% (MTD); LOS: 19 vs. 17 days; CC0/CC1 resection: 73.8%	5-year OS: 87.4% (CCRS) vs. 39.2% (MTD); Byrne cohort 5-year OS: 65.6%	NNT: 2 patients	A (High quality)	Refer eligible Stage IV mucinous patients to specialized centers for CRS/HIPEC evaluation	<0.001
Chemotherapy effectiveness by histology	8733 patients (Wang et al., 2021) [31]	Chemotherapy benefit: MAC vs. NMAC vs. SRCC	MAC: 0.85 (0.76–0.95); NMAC: 0.92 (0.84–1.01); SRCC: 1.15 (0.98–1.35)	Histology-dependent chemotherapy effectiveness (MAC > NMAC > SRCC)	Selection bias evident in overall chemotherapy studies	5-year OS with chemotherapy: MAC 65.8%, NMAC 56.2%, SRCC 48.2%	NNT: MAC 10 patients; NMAC 26 patients; SRCC no benefit	B (High quality for MAC, moderate for others)	Prioritize chemotherapy for MAC, consider for NMAC, limited benefit in SRCC	MAC: 0.003; NMAC: 0.08; SRCC: 0.12
Goblet cell adenocarcinoma	2701 patients (El Asmar et al., 2024) [24]	Surgical approach for GCA	0.75 (0.65–0.87)	Favorable prognosis with appropriate surgical management	Geographic variation: UK vs. US outcomes	5-year OS: 73.8% (UK) vs. 79.6% (US)	Generally favorable outcomes	B (Moderate quality)	Right hemicolectomy with lymph node dissection regardless of size	<0.05
Well-differentiated neuroendocrine tumors	5655 patients (Turaga et al., 2012) [38]; 2891 patients (Wang et al., 2023) [30]	Size-based surgical approach	0.25 (0.18–0.35)	Excellent prognosis with appropriate surgery	Low operative mortality for appendectomy approach	5-year OS: 93% (well-differentiated carcinoid)	Favorable histology—minimize morbidity	B (Moderate quality)	Appendectomy for <2 cm, right hemicolectomy for ≥2 cm or poorly differentiated	<0.001
Signet ring cell carcinoma	Multiple studies (Wang et al., 2021; Turaga et al., 2012) [31,38]	Aggressive vs. conservative surgical approach	1.85 (1.45–2.36)	Poor prognosis despite aggressive treatment	High mortality regardless of approach	5-year OS: 27–48.2% across studies	Consider experimental approaches	C (Low quality—rare subtype)	Aggressive surgical resection, consider clinical trials and experimental therapy	<0.001
Elderly patients (>70 years) with comorbidities	Subset analysis from Marks et al., 2023 [23]	Extensive vs. limited surgical approach	2.15 (1.75–2.64)	Higher baseline mortality with extensive surgery	Comorbidity-adjusted mortality rates significantly higher	Individualized based on functional status and life expectancy	Risk-benefit individualization needed	C (Expert consensus)	Individualized approach prioritizing quality of life and functional status	<0.01

**Abbreviations:** CC, completeness of cytoreduction; CCRS, complete cytoreductive surgery; CI, confidence interval; CRS, cytoreductive surgery; GCA, goblet cell adenocarcinoma; hemi, hemicolectomy; HIPEC, hyperthermic intraperitoneal chemotherapy; HR, hazard ratio; LOS, length of stay; LVI, lymphovascular invasion; MAC, mucinous adenocarcinoma; MTD, maximum tolerated dose; NHB, non-Hispanic Black; NHW, non-Hispanic White; NMAC, non-mucinous adenocarcinoma; NNH, number needed to harm; NNT, number needed to treat; OS, overall survival; RHC, right hemicolectomy; SRCC, signet ring cell carcinoma; UK, United Kingdom; US, United States.

**Table 5:** Quality metrics of predictive modeling, model performance, and key findings.

Study Name	Database	Sample Size	Patient Population	30-Day Mortality	90-Day Mortality	Readmission Rate	Length of Stay	Quality Predictors	Risk Stratification	Model Performance	Key Findings
Baron et al., 2024 [25]	NCDB	6800	Stage I–III appendiceal adenocarcinoma	RM−: 1.1%; RM+: 3.7%	RM−: 1.7%; RM+: 6.9%	RM−: 4.2%; RM+: 5.6%	RM−: 5 days; RM+: 6 days	Resection margin status (OR: 3.4 for 30-day mortality)	Low risk (RM−): 1.1–1.7%; High risk (RM+): 3.7–6.9%	Margin status most discriminative predictor	Positive margins increase 30-day mortality 3.4×, 90-day mortality 4.1×
Emile et al., 2024 [28]	NCDB	2607	Stage I–III appendiceal adenocarcinoma	Hemicolectomy: 1.8%; Partial: 2.8%	Hemicolectomy: 3.3%; Partial: 4.2%	Hemicolectomy: 5%; Partial: 4%	5 days (both procedures)	Surgical approach (OR: 1.6), LVI status (21.1% prevalence)	Low risk (Hemi): 1.8–3.3%; High risk (Partial): 2.8–4.2%	Surgical approach primary predictor	Hemicolectomy reduces 30-day mortality 1.6×, 90-day mortality 1.3× vs. partial colectomy
Freudenberger et al., 2023 [29]	NCDB	2532	CRS/HIPEC patients	NHW: 0.9%; NHB: 2.2%	NHW: 2.8%; NHB: 2.7%	NHW: 6.2%; NHB: 7.5%	9 days (both groups)	Race/ethnicity (OR: 2.4 for NHB), positive margins: 9.3%	Low risk (NHW): 0.9–2.8%; High risk (NHB): 2.2–7.5%	Race strongest predictor in HIPEC patients	NHB patients have 2.4× higher 30-day mortality, 1.2× higher readmissions
Marks et al., 2023 [23]	NCDB	18,216	All appendiceal neoplasm types	Appendectomy: 0.9%; RHC: 1.4%	Appendectomy: 1.5%; RHC: 3.2%	Appendectomy: 3%; RHC: 5%	Appendectomy: 3 days; RHC: 5 days	Surgical extent (OR: 1.6 for RHC vs. appendectomy)	Low risk (Appendectomy): 0.9–3%; High risk (RHC): 1.4–5%	Surgical complexity strongest predictor	RHC increases 30-day mortality 1.6×, 90-day mortality 2.1×, readmissions 1.7×
Ansari et al., 2016 [37]	Institutional	1000	CRS/HIPEC patients	CCRS: 0.8%; MTD: 1.7%	NR	NR	CCRS: 19 days; MTD: 17 days	Completeness of cytoreduction (CC0/CC1: 73.8%)	Low risk (CCRS): 0.8%; High risk (MTD): 1.7%	Complete cytoreduction primary predictor	Complete cytoreduction achieves lowest mortality rates despite complex procedures

**Abbreviations:** CCRS, complete cytoreductive surgery; CRS, cytoreductive surgery; HIPEC, hyperthermic intraperitoneal chemotherapy; LVI, lymphovascular invasion; MTD, maximum tolerated dose; NCDB, National Cancer Database; NHB, non-Hispanic Black; NHW, non-Hispanic White; NR, not reported; OR, odds ratio; RHC, right hemicolectomy; RM, resection margin.

## Data Availability

The original contributions presented in this study are included in the article/Supplementary Materials. Further inquiries can be directed to the corresponding author.

## Supplementary Materials

The following supporting information can be downloaded at: https://www.mdpi.com/article/10.3390/healthcare13233074/s1, Table S1: Population Phenotyping Discovery and Treatment Response Evaluation and Assessment; Table S2: Epidemiological Trends and Future Disease Burden Projections; Table S3: Advanced Statistical Methodology, Validation, Uncertainty Quantification, and Evidence Quality Assessment Using Modified Newcastle-Ottawa Scale; Figure S1: Distribution of Reconstructed Patient-Level Covariates; Figure S2: Study Overlap Network; Figure S3: Pairwise Overlap Probability Matrix; Figure S4: Propensity Score Analysis for HIPEC Treatment; Figure S5: Multi-Level Model Validation Framework; Figure S6: Variable Preprocessing and Standardization; Figure S7: Validation of Kaplan-Meier Curve Reconstruction; Figure S8: Bootstrap Distribution Analysis; Figure S9: Model Calibration Across Time Horizons; Figure S10: Time-Dependent ROC Curves for Risk Stratification Model.
