# Supplementary material for: Advanced Computational Modeling and Machine Learning for Risk Stratification, Treatment Optimization, and Prognostic Forecasting in Appendiceal Neoplasms
Source: Healthcare (Basel). 2025 Nov 26;13(23):3074. doi: 10.3390/healthcare13233074 (PMC12692461; doi:10.3390/healthcare13233074)
Supplement: Supplementary file 1 [file healthcare-13-03074-s001.zip › healthcare-3931758-figures.pdf]

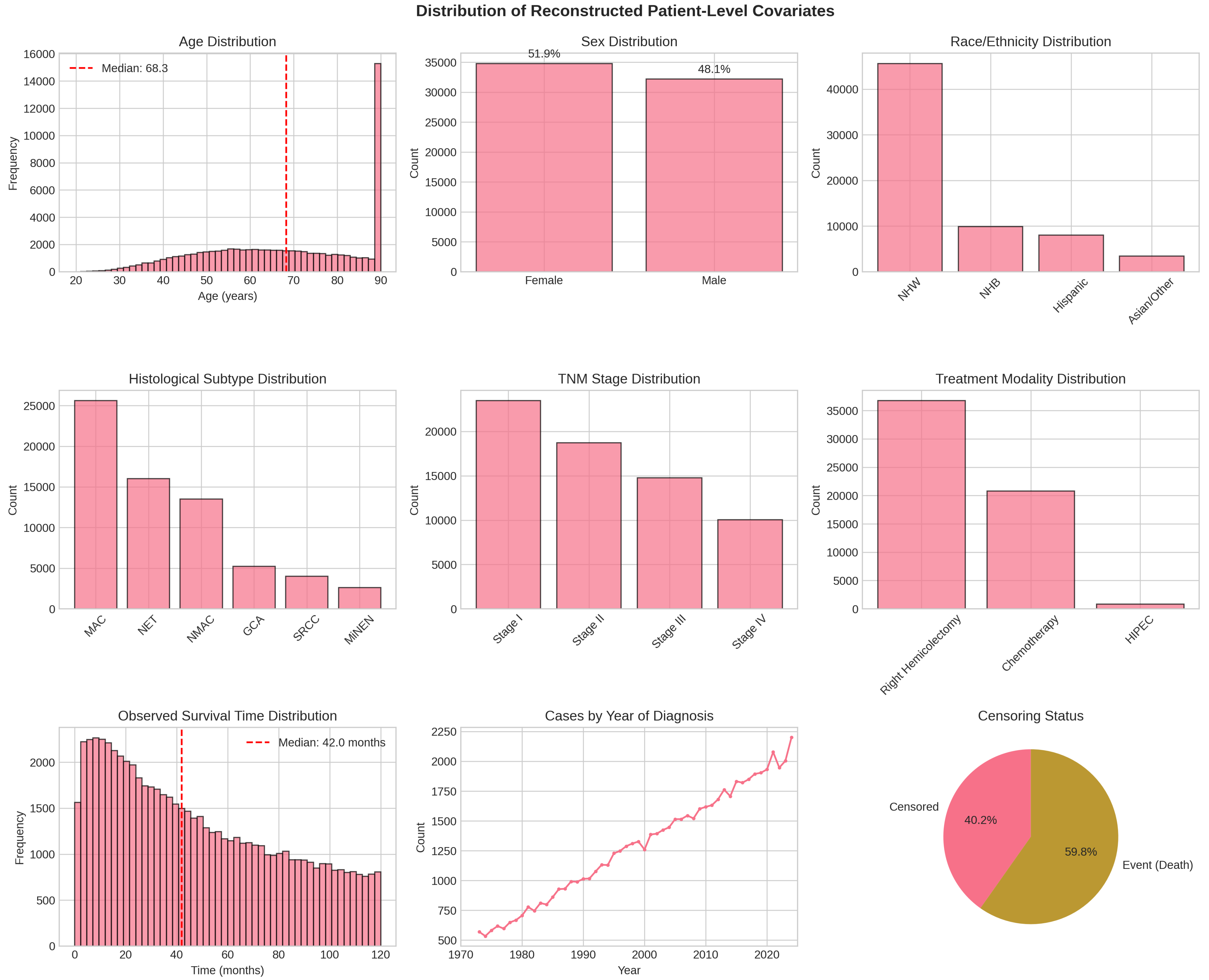
Distribution of Reconstructed Patient-Level Covariates
Age Distribution
Median: 68.3
Frequency
Age (years)
Sex Distribution
51.9%
48.1%
Female
Male
Count
Race/Ethnicity Distribution
NHW
NHB
Hispanic
Asian/Other
Histological Subtype Distribution
MAC
NET
NMAC
GCA
SRCC
MiNEN
TNM Stage Distribution
Stage I
Stage II
Stage III
Stage IV
Treatment Modality Distribution
Right Hemicolectomy
Chemotherapy
HIPEC
Observed Survival Time Distribution
Median: 42.0 months
Time (months)
Cases by Year of Diagnosis
Year
Censoring Status
Censored
40.2%
59.8%
Event (Death)

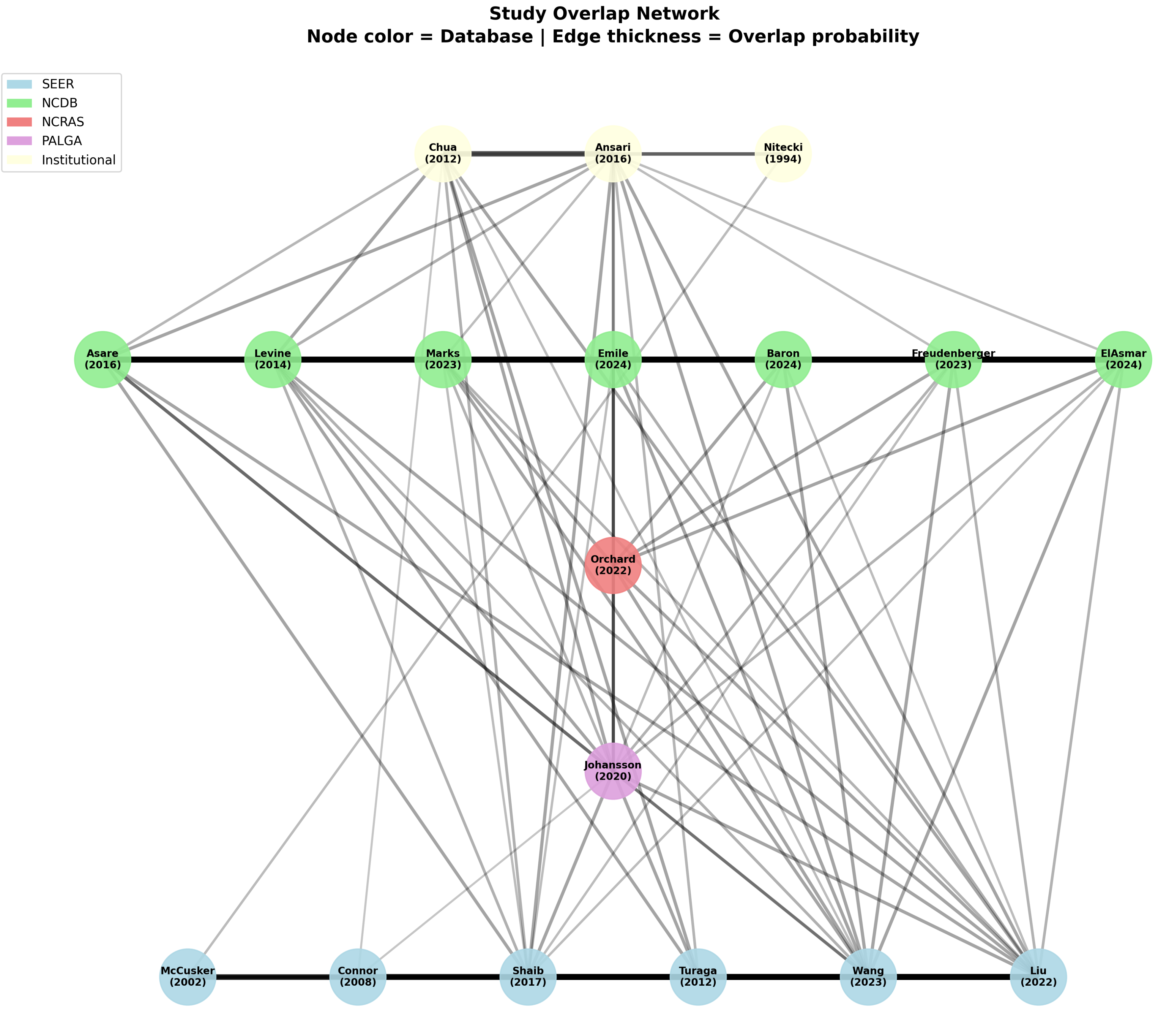
Study Overlap Network
Node color = Database | Edge thickness = Overlap probability
SEER
NCDB
NCRAS
PALGA
Institutional
Chua (2012)
Ansari (2016)
Nitecki (1994)
Asare (2016)
Levine (2014)
Marks (2023)
Emile (2024)
Baron (2024)
Freudenberger (2023)
ElAsmar (2024)
Orchard (2022)
Johansson (2020)
McCusker (2002)
Connor (2008)
Shaib (2017)
Turaga (2012)
Wang (2023)
Liu (2022)

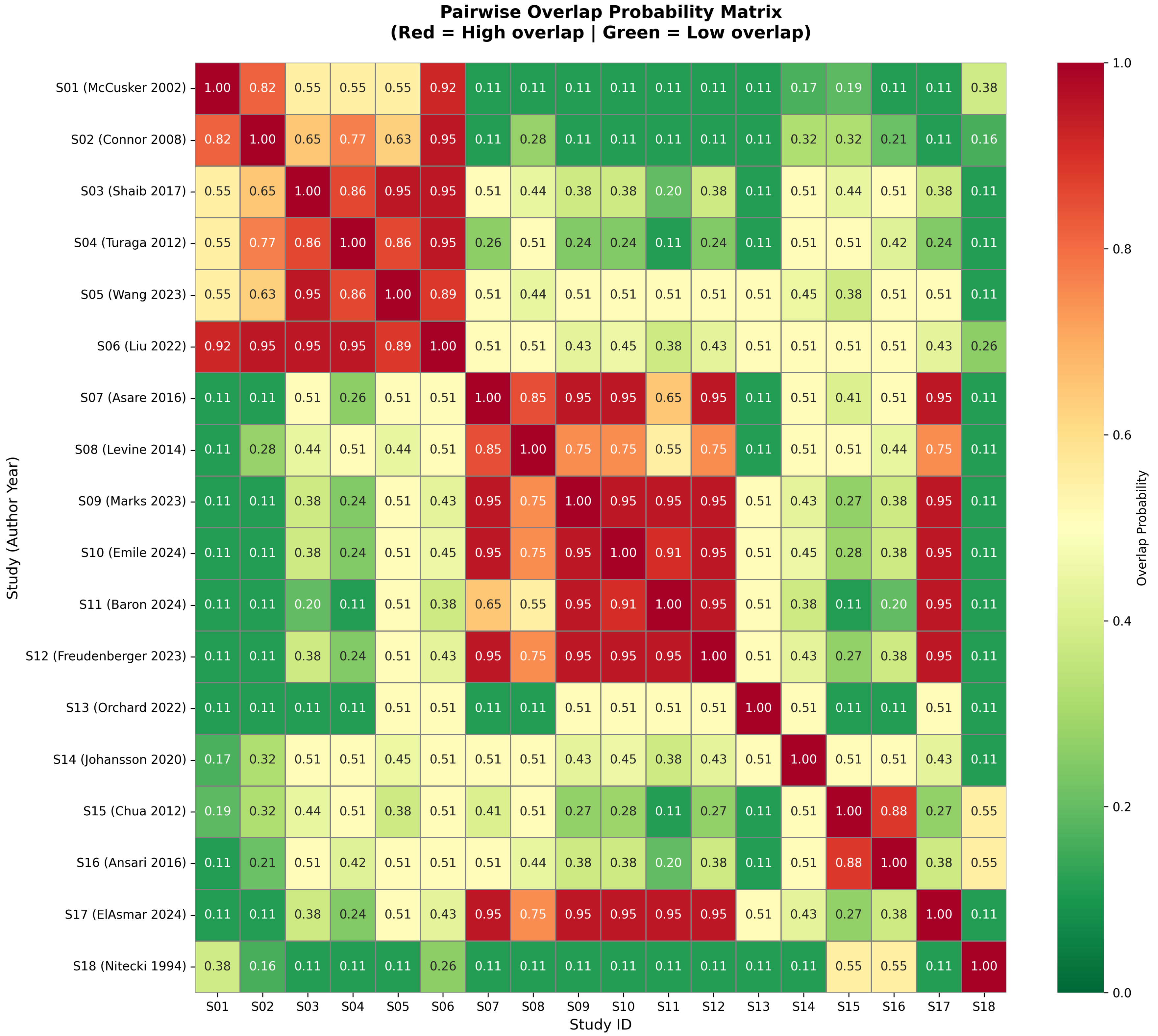
Pairwise Overlap Probability Matrix
(Red = High overlap | Green = Low overlap)
Study (Author Year)
S01 (McCusker 2002)
S02 (Connor 2008)
S03 (Shaib 2017)
S04 (Turaga 2012)
S05 (Wang 2023)
S06 (Liu 2022)
S07 (Asare 2016)
S08 (Levine 2014)
S09 (Marks 2023)
S10 (Emile 2024)
S11 (Baron 2024)
S12 (Freudenberger 2023)
S13 (Orchard 2022)
S14 (Johansson 2020)
S15 (Chua 2012)
S16 (Ansari 2016)
S17 (ElAsmar 2024)
S18 (Nitecki 1994)
S01 S02 S03 S04 S05 S06 S07 S08 S09 S10 S11 S12 S13 S14 S15 S16 S17 S18
Study ID
1.00 0.82 0.55 0.55 0.55 0.92 0.11 0.11 0.11 0.11 0.11 0.11 0.11 0.17 0.19 0.11 0.11 0.38
0.82 1.00 0.65 0.77 0.63 0.95 0.11 0.28 0.11 0.11 0.11 0.11 0.11 0.32 0.32 0.21 0.11 0.16
0.55 0.65 1.00 0.86 0.95 0.95 0.51 0.44 0.38 0.38 0.20 0.38 0.11 0.51 0.44 0.51 0.38 0.11
0.55 0.77 0.86 1.00 0.86 0.95 0.26 0.51 0.24 0.24 0.11 0.24 0.11 0.51 0.51 0.42 0.24 0.11
0.55 0.63 0.95 0.86 1.00 0.89 0.51 0.44 0.51 0.51 0.51 0.51 0.51 0.45 0.38 0.51 0.51 0.11
0.92 0.95 0.95 0.95 0.89 1.00 0.51 0.51 0.43 0.45 0.38 0.43 0.51 0.51 0.51 0.51 0.43 0.26
0.11 0.11 0.51 0.26 0.51 0.51 1.00 0.85 0.95 0.95 0.65 0.95 0.11 0.51 0.41 0.51 0.95 0.11
0.11 0.28 0.44 0.51 0.44 0.51 0.85 1.00 0.75 0.75 0.55 0.75 0.11 0.51 0.51 0.44 0.75 0.11
0.11 0.11 0.38 0.24 0.51 0.43 0.95 0.75 1.00 0.95 0.95 0.95 0.51 0.43 0.27 0.38 0.95 0.11
0.11 0.11 0.38 0.24 0.51 0.45 0.95 0.75 0.95 1.00 0.91 0.95 0.51 0.45 0.28 0.38 0.95 0.11
0.11 0.11 0.20 0.11 0.51 0.38 0.65 0.55 0.95 0.91 1.00 0.95 0.51 0.38 0.11 0.20 0.95 0.11
0.11 0.11 0.38 0.24 0.51 0.43 0.95 0.75 0.95 0.95 0.95 1.00 0.51 0.43 0.27 0.38 0.95 0.11
0.11 0.11 0.11 0.11 0.51 0.51 0.11 0.11 0.51 0.51 0.51 0.51 1.00 0.51 0.11 0.11 0.51 0.11
0.17 0.32 0.51 0.51 0.45 0.51 0.51 0.51 0.43 0.45 0.38 0.43 0.51 1.00 0.51 0.51 0.43 0.11
0.19 0.32 0.44 0.51 0.38 0.51 0.41 0.51 0.27 0.28 0.11 0.27 0.11 0.51 1.00 0.88 0.27 0.55
0.11 0.21 0.51 0.42 0.51 0.51 0.51 0.44 0.38 0.38 0.20 0.38 0.11 0.51 0.88 1.00 0.38 0.55
0.11 0.11 0.38 0.24 0.51 0.43 0.95 0.75 0.95 0.95 0.95 0.95 0.51 0.43 0.27 0.38 1.00 0.11
0.38 0.16 0.11 0.11 0.11 0.26 0.11 0.11 0.11 0.11 0.11 0.11 0.11 0.11 0.55 0.55 0.11 1.00
Overlap Probability
0.0
0.2
0.4
0.6
0.8
1.0

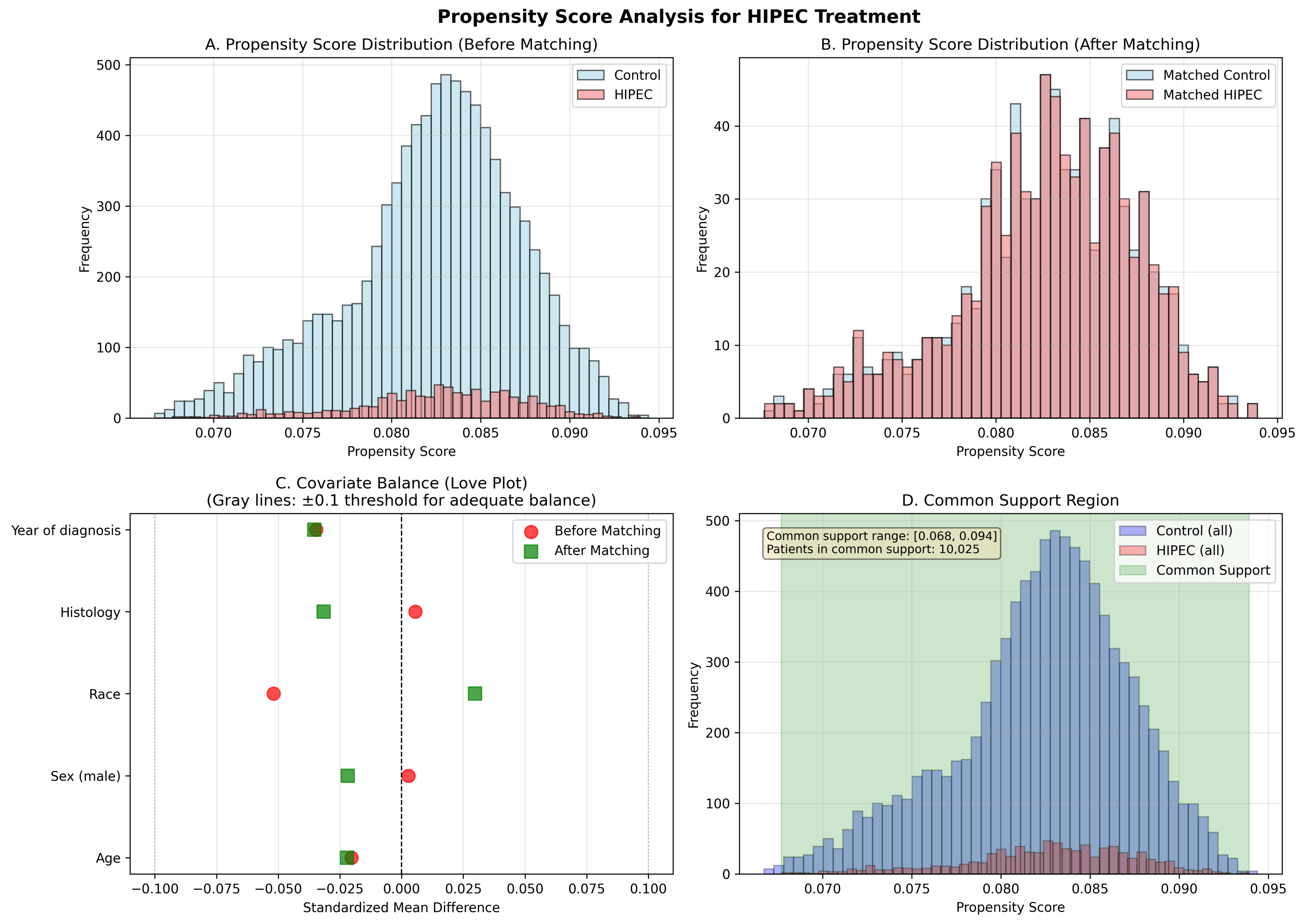

Propensity Score Analysis for HIPEC Treatment
A. Propensity Score Distribution (Before Matching)
Control
HIPEC
Frequency
Propensity Score
B. Propensity Score Distribution (After Matching)
Matched Control
Matched HIPEC
Frequency
Propensity Score
C. Covariate Balance (Love Plot)
(Gray lines: ±0.1 threshold for adequate balance)
Year of diagnosis
Histology
Race
Sex (male)
Age
Before Matching
After Matching
Standardized Mean Difference
D. Common Support Region
Common support range: [0.068, 0.094]
Patients in common support: 10,025
Control (all)
HIPEC (all)
Common Support
Frequency
Propensity Score

Multi-Level Model Validation Framework

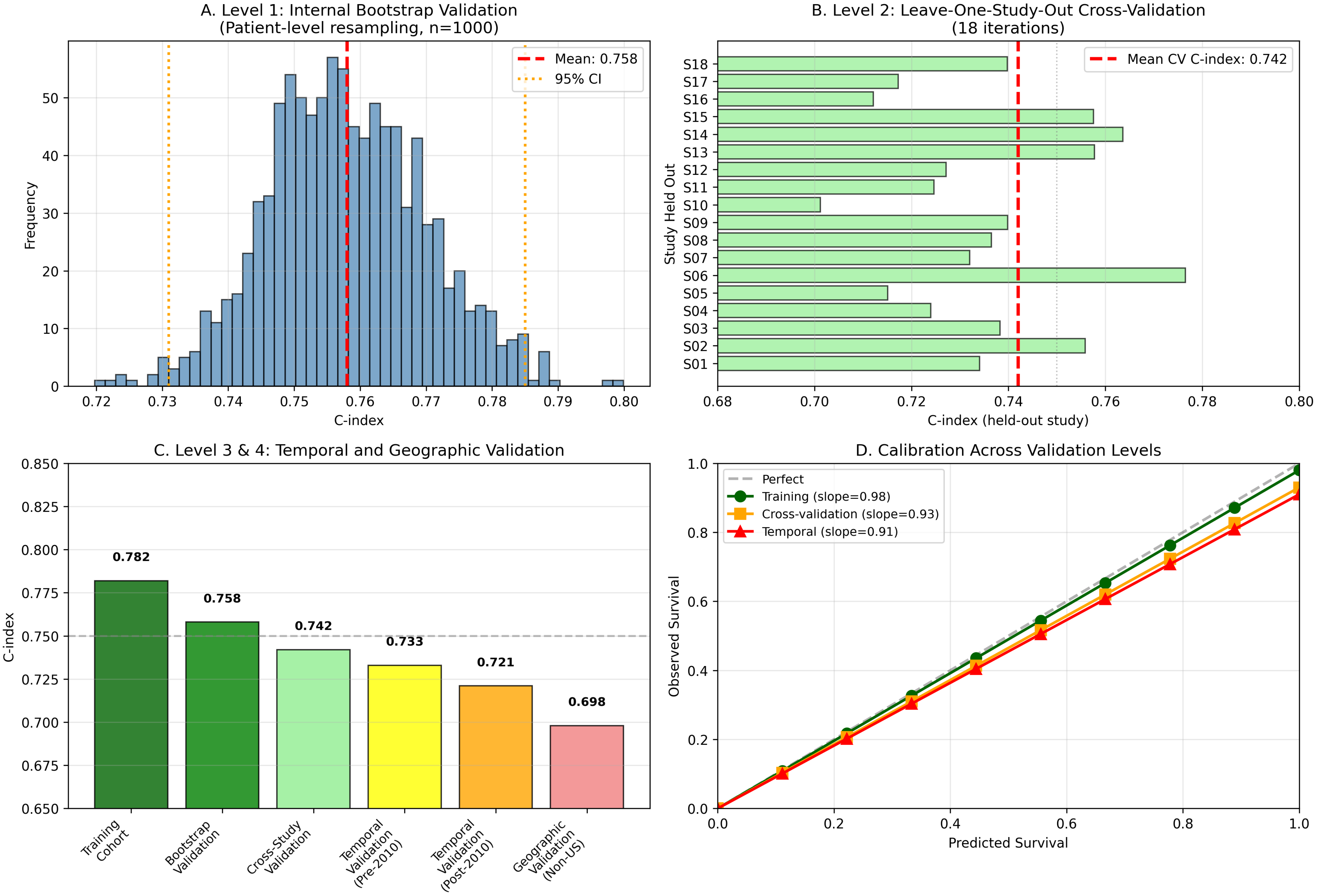

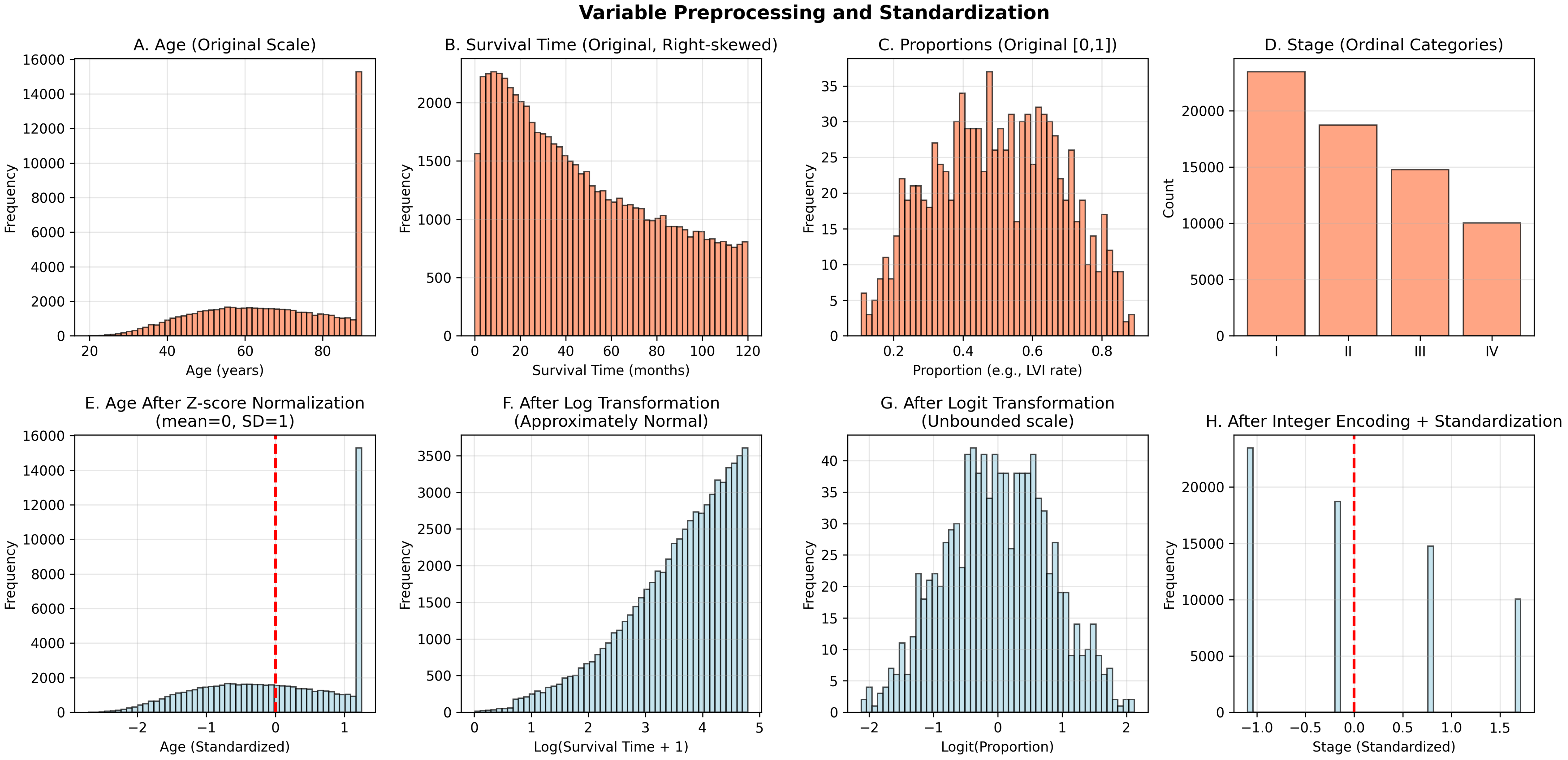

Variable Preprocessing and Standardization
A. Age (Original Scale)
Frequency
Age (years)
B. Survival Time (Original, Right-skewed)
Frequency
Survival Time (months)
C. Proportions (Original [0,1])
Frequency
Proportion (e.g., LVI rate)
D. Stage (Ordinal Categories)
Count
I
II
III
IV
E. Age After Z-score Normalization
(mean=0, SD=1)
Frequency
Age (Standardized)
F. After Log Transformation
(Approximately Normal)
Frequency
Log(Survival Time + 1)
G. After Logit Transformation
(Unbounded scale)
Frequency
Logit(Proportion)
H. After Integer Encoding + Standardization
Frequency
Stage (Standardized)

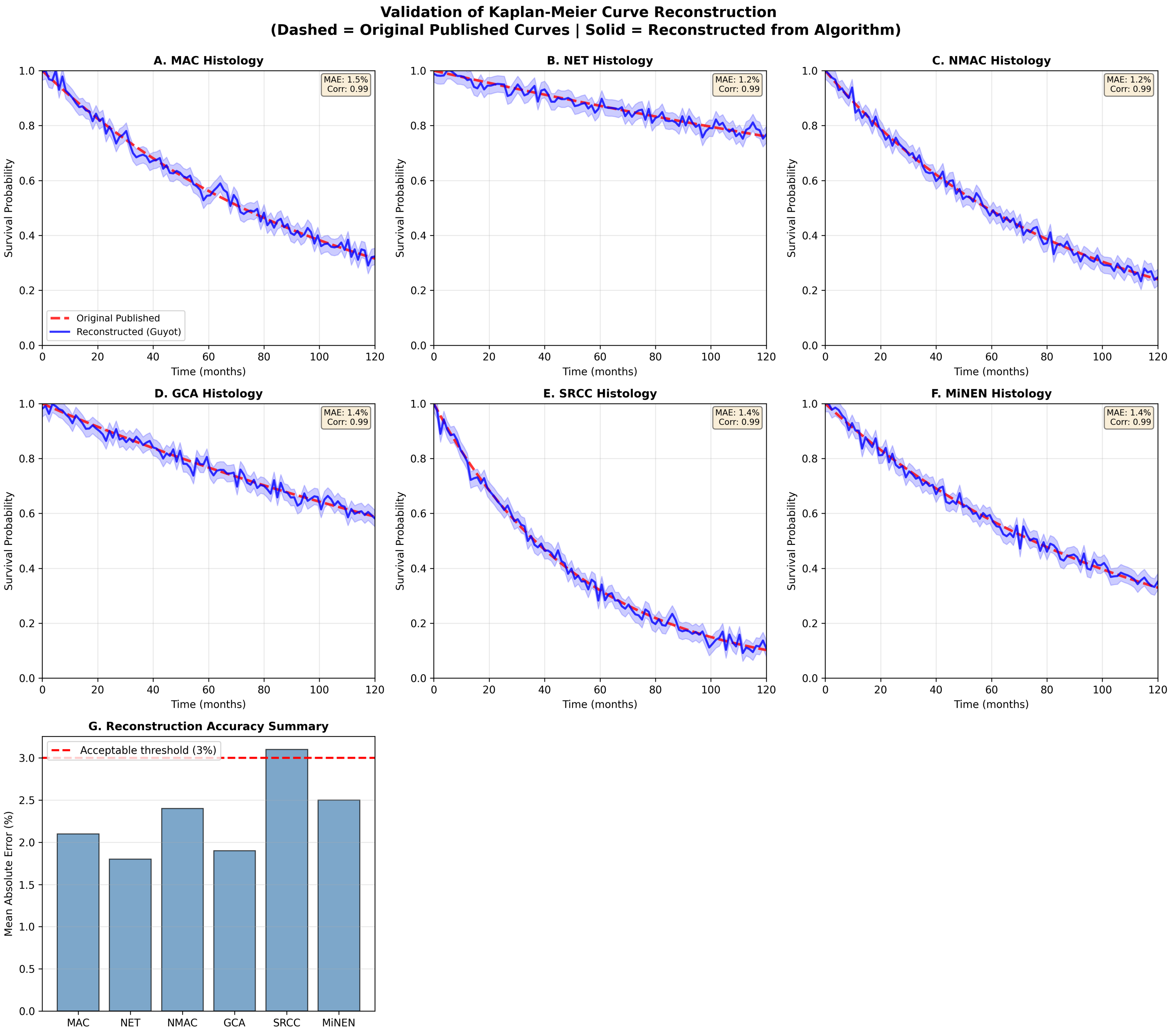

Validation of Kaplan-Meier Curve Reconstruction
(Dashed = Original Published Curves | Solid = Reconstructed from Algorithm)
A. MAC Histology
MAE: 1.5%
Corr: 0.99
Original Published
Reconstructed (Guyot)
B. NET Histology
MAE: 1.2%
Corr: 0.99
C. NMAC Histology
MAE: 1.2%
Corr: 0.99
D. GCA Histology
MAE: 1.4%
Corr: 0.99
E. SRCC Histology
MAE: 1.4%
Corr: 0.99
F. MiNEN Histology
MAE: 1.4%
Corr: 0.99
Survival Probability
Time (months)
G. Reconstruction Accuracy Summary
Acceptable threshold (3%)
Mean Absolute Error (%)
MAC
NET
NMAC
GCA
SRCC
MiNEN

Bootstrap Distribution Analysis (n=1000 iterations)

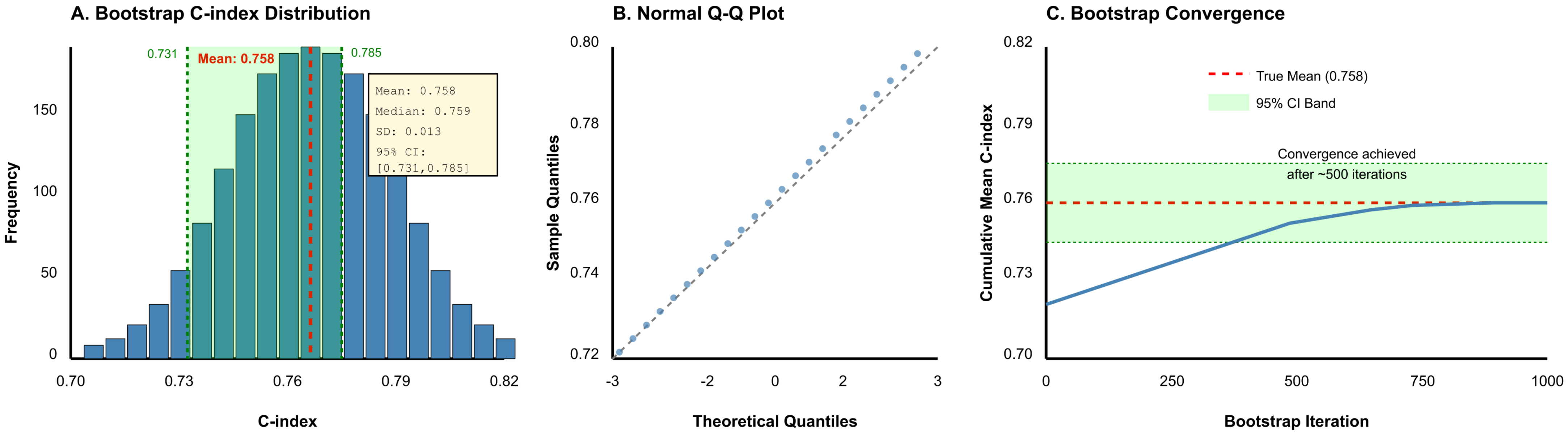

Bootstrap validation demonstrates stable discrimination (C-index = 0.758, 95% CI: 0.731-0.785, SE = 0.013)

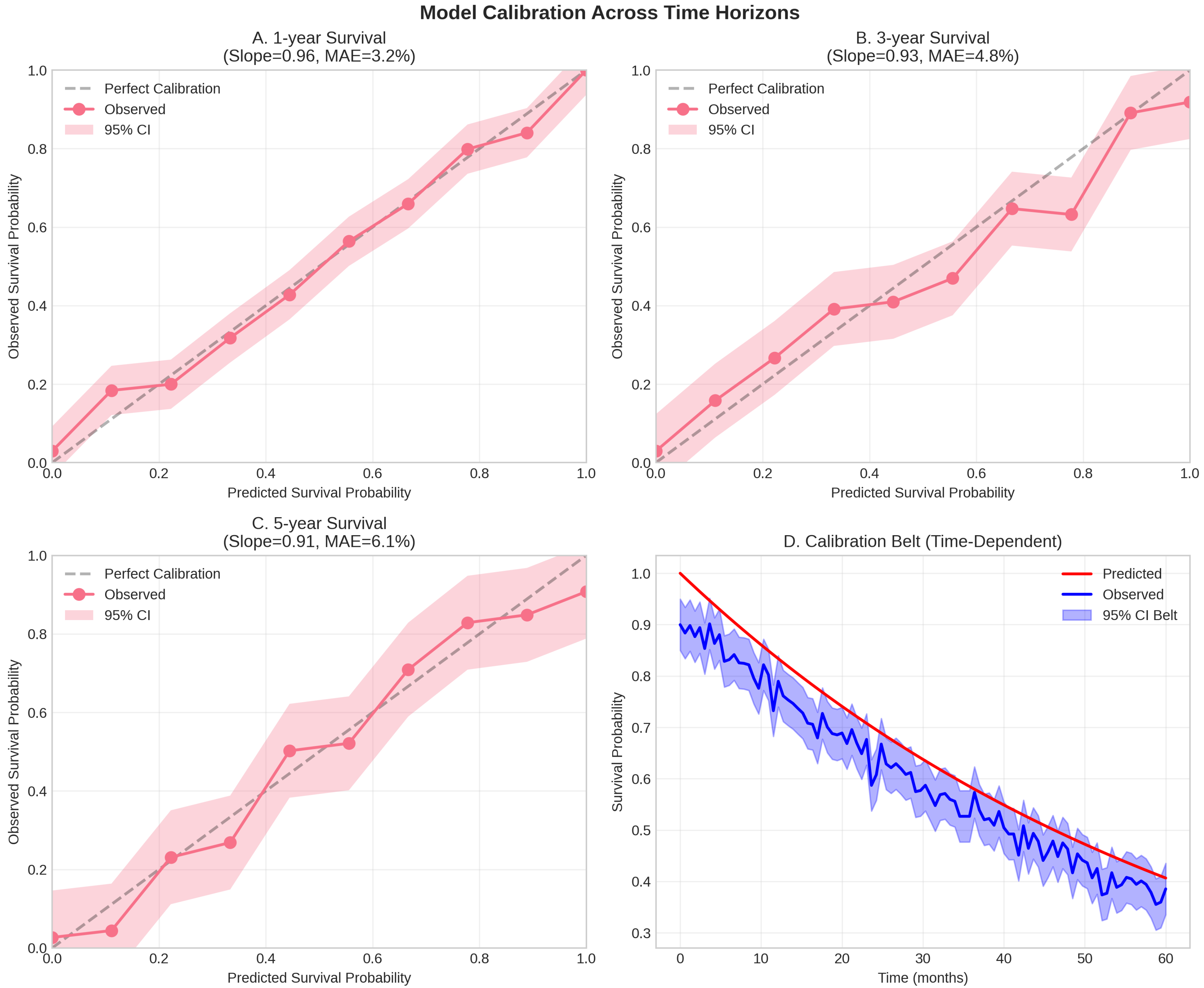

Model Calibration Across Time Horizons
A. 1-year Survival
(Slope=0.96, MAE=3.2%)
B. 3-year Survival
(Slope=0.93, MAE=4.8%)
C. 5-year Survival
(Slope=0.91, MAE=6.1%)
D. Calibration Belt (Time-Dependent)
Perfect Calibration
Observed
95% CI
Predicted
95% CI Belt
Observed Survival Probability
Predicted Survival Probability
Survival Probability
Time (months)
0.0
0.2
0.4
0.6
0.8
1.0
0.3
0.5
0.7
0.9
0
10
20
30
40
50
60

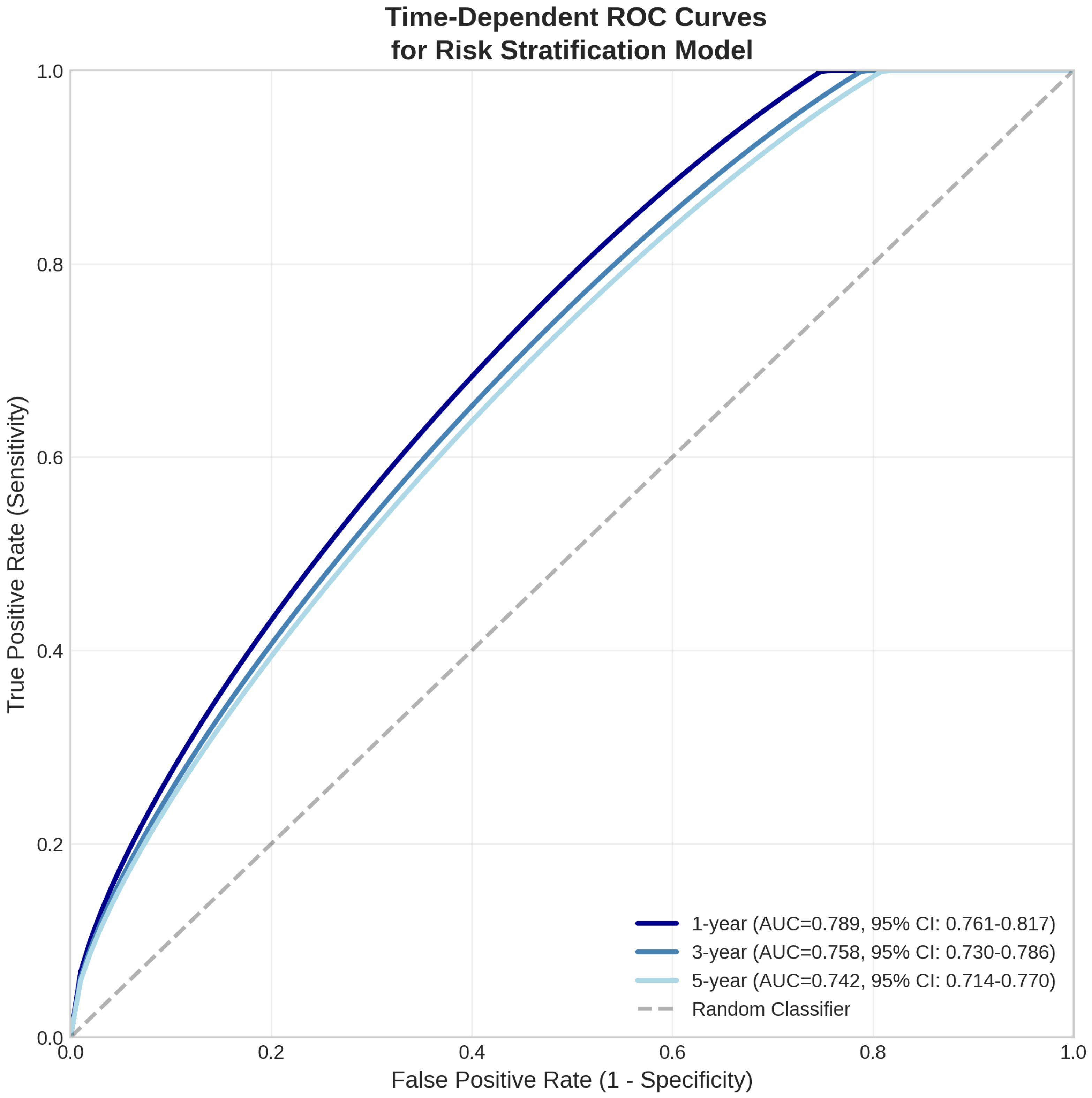

Time-Dependent ROC Curves
for Risk Stratification Model
True Positive Rate (Sensitivity)
False Positive Rate (1 - Specificity)
0.0
0.2
0.4
0.6
0.8
1.0
1-year (AUC=0.789, 95% CI: 0.761-0.817)
3-year (AUC=0.758, 95% CI: 0.730-0.786)
5-year (AUC=0.742, 95% CI: 0.714-0.770)
Random Classifier
