# Supplementary material for: Advanced Computational Modeling and Machine Learning for Risk Stratification, Treatment Optimization, and Prognostic Forecasting in Appendiceal Neoplasms
Source: Healthcare (Basel). 2025 Nov 26;13(23):3074. doi: 10.3390/healthcare13233074 (PMC12692461; doi:10.3390/healthcare13233074)
Supplement: Supplementary file 1 [file healthcare-13-03074-s001.zip › healthcare-3931758-supplementary.v5.pdf]

Supplementary Table S1: Population Phenotyping Discovery and Treatment Response Evaluation and Assessment.

Patient Phenotype	Included Studies	Total Sample Size	Demographic Characteristics	Histological Profile	Treatment Patterns	Quality Metrics	Survival Outcomes	Prognostic Factors	Surgical Treatment Response	Chemotherapy Response	HIPEC Utilization	Validation	Recommendations	Evidence Level	Utilization	Decision Support Applications	Future Research Directions	Significance
High-Quality Surgical Outcome Cohort	Baron et al. 2024 [25]; Emile et al. 2024 [28] ; Freudenberger et al. 2023 [29] ; Marks et al. 2023 [23]	30,155	Age 57-62 years, Balanced sex distribution (47-52% male)	Mixed adenocarcinoma (MAC: 34-46%, NMAC: 24-50%, SRCC: 7-9%)	Surgical resection focus (RHC: 60-72%, Chemotherapy: 37-39%)	30-day mortality: 0.9-2.8%, 90-day mortality: 1.5-4.2%, LOS: 3-9 days	Median OS: 54-136 months, 5-year OS: 58%	Margin status significant predictor (RM+ vs RM-)	RHC preferred (60-72%), Associated with improved margin control	Adjuvant benefit demonstrated (37-39% utilization)	Selected cases (Freudenberger: 100% in specialized series)	Cross-validated across 4 large NCDB studies (n=30,155)	RHC for resectable disease, Adjuvant chemotherapy for high-risk features, Quality metrics monitoring	Level I (Multiple large cohort studies with quality metrics)	Quality improvement benchmarking, Operative planning, Risk stratification	Pre-operative mortality risk assessment, Post-operative monitoring protocols	Quality metric standardization, Margin optimization studies	Representative of modern US cancer care quality metrics
Population-Based Epidemiological Cohort	Wang et al. 2023 [30]; Wang et al. 2021 [31]; Zheng et al. 2020 [32]; Yan et al. 2019 [34]; Shaib et al. 2017 [35] ; Xie et al. 2016 [21] ; Marmor et al. 2015 [13] ; Turaga et al. 2012 [38] ; McCusker et al. 2002 [40]	38,323	Age 46-62 years, Slight female predominance (45-50% male)	Histologic diversity (MAC: 26-100%, NET: 11-47%, Variable SRCC/GCA)	Variable approaches (RHC: 36-70%, Appendectomy: 2-44%, Chemo: 31-47%)	Limited quality data reported	5-year OS: 48-93% (histology-dependent), Median OS: 42-85 months	Histology type primary predictor (NET > MAC > SRCC)	Variable approaches (RHC: 36-70%, Appendectomy: 2-44%)	Histology-dependent benefit (NET: Limited, MAC: Moderate)	Rare utilization in population studies	Validated across 9 SEER studies spanning 1973-2020 (n=38,323)	Histology-guided treatment selection, NET: Surgery primary, MAC: Multimodal approach, SRCC: Aggressive therapy	Level I (Large population-based registries, long-term follow-up)	Natural history understanding, Treatment effectiveness assessment, Guideline development	Histology-based treatment algorithms, Prognosis communication	Long-term outcome studies, Treatment evolution analysis	Population-level trends and natural history understanding
Advanced Specialized Treatment Cohort	Ansari et al. 2016 [37] ; El Asmar et al. 2024 [24]	3701	Age 56-58 years, Variable sex distribution	Specialized focus (GCA: 100% El Asmar, Mixed Ansari)	Specialized approaches (HIPEC: 100%, International comparison)	Enhanced surgical metrics (CC scores, specialized LOS)	Superior outcomes (5-year OS: 74-87% specialized care)	Complete cytoreduction (CC score) primary predictor	Complete cytoreduction focus (CC-0/CC-1: 74%)	Integrated with HIPEC protocols	Standard approach (100% in specialized centers)	Validated in specialized centers (UK and tertiary institutions)	HIPEC for appropriate candidates, Complete cytoreduction priority, Multidisciplinary team approach	Level II (Specialized institutional series with enhanced outcomes)	HIPEC candidate selection, Specialized center outcomes, International benchmarking	Complex case management, Referral decision algorithms	HIPEC optimization studies, International protocol standardization	Benchmark for specialized care and international comparisons
Historical Comparison Cohort	Asare et al. 2016 [36] ; Smeenk et al. 2008 [39]	13,353	Age 58-64 years, Balanced demographics	Traditional classification (MAC vs Non-mucinous focus)	Era-specific treatment patterns (Lower HIPEC utilization)	Limited contemporary quality metrics	Mucinous vs non-mucinous survival differences	Mucinous histology associated with distinct outcomes	Era-dependent approaches, Lower HIPEC utilization	Mucinous vs non-mucinous differential benefit	Limited historical utilization	International validation (US and European registries)	Mucinous subtype-specific protocols, Era-adjusted outcome expectations	Level II (Historical cohorts with international validation)	Treatment evolution documentation, International comparison, Subtype-specific insights	Era-adjusted prognostic models, Comparative effectiveness assessment	Long-term trend analysis, International guideline harmonization	Historical context and international validation

Abbreviations: CC, completeness of cytoreduction; GCA, goblet cell adenocarcinoma; HIPEC, hyperthermic intraperitoneal chemotherapy; LOS, length of stay; MAC, mucinous adenocarcinoma; NCDB, National Cancer Database; NET, neuroendocrine tumor; NMAC, non-mucinous adenocarcinoma; OS, overall survival; RHC, right hemicolectomy; RM, resection margin; SEER, Surveillance, Epidemiology, and End Results; SRCC, signet ring cell carcinoma; UK, United Kingdom; US, United States.

Supplementary Table S2: Epidemiological Trends and Future Disease Burden Projections.

**Time Period**	**Study/Projection Domain**	**Database/Scenario**	**Sample Size/Scale**	**Histology Evolution (%)**	**Stage Distribution Trends**	**Treatment Pattern Changes**	**Survival Improvements**	**Incidence Trends (per 100,000)**	**Implications**
1973-1998	McCusker et al. 2002 [38]	SEER	1645	MAC: 37.3; NMAC: 25.0; SRCC: 4.3; GCA: 13.8; NET: 19.7	Local/Regional: 37% (MAC); Distant: 63% (MAC)	RHC: 52% (MAC); Less extensive surgery: 38%	Variable by histology and stage	0.012 (Stable trend)	Baseline reference period
1973-2007	Turaga et al. 2012 [38]	SEER	5655	MAC: 37.0; NMAC: 27.0; SRCC: 5.5; GCA: 19.0; NET: 11.0	Mixed distribution across stages	RHC: 39%; Partial colectomy: 32%	5-year OS: 93% (NET), 58% (MAC), 27% (SRCC)	0.2-0.6 (Increasing)	Early evidence of increasing incidence
1985-2006	Asare et al. 2016 [36]	NCDB	11,871	MAC: 50.3; NMAC: 40.5; SRCC: 9.2	Stage IV: 52% (MUC) vs 26.2% (NON-MUC)	Chemotherapy: 51.8% (MUC), 39.8% (NON-MUC)	5-year OS: 53.6% (MUC), 46.2% (NON-MUC)	NR	Histology-dependent staging patterns
1973-2011	Shaib et al. 2017 [35]	SEER	2733	MAC: 100% (mucinous focus)	Localized: 26.3%; Regional: 20.5%; Distant: 53.2%	RHC: 70.6%; Appendectomy: 2.2%	Median OS: 42 months (distant stage)	0.6-2.8 (Increasing)	Confirmed increasing trend
1973-2015	Yan et al. 2019 [34]	SEER	3237	MAC: 100% (mucinous adenocarcinoma focus)	Stage I-II: 13.5%; Combined III-IV: 46.4%	RHC: 36%; Appendectomy: 25.2%; Chemotherapy: 47%	Median OS: 80 months; 5-year OS: 56.2%	0.05-0.25 (Increasing)	Long-term trend validation
2000-2009	Marmor et al. 2015 [13]	SEER	4765	MAC: 38.0; NMAC: 27.0; SRCC: 7.0; NET: 28.0	Localized: 26%; Regional: 39%; Distant: 35%	Treatment patterns evolving	5-year OS: 77% (local), 60% (regional), 33% (distant)	0.63-0.97 (Increasing)	Stage-dependent outcome stratification
1998-2016	Wang et al. 2021 [31]	SEER	8733	MAC: 32.4; NMAC: 20.2; SRCC: 6.6; GCA: 12.5; NET: 23.9	Variable across histologic types	RHC: 50.5%; Appendectomy: 44.1%; Chemotherapy: 31.8%	5-year OS: 65.8% (MAC), 56.2% (NMAC), 48.2% (SRCC)	NR	Histology-specific survival patterns
2004-2015	Wang et al. 2023 [30]	SEER	2891	MAC: 25.6; NMAC: 21.4; SRCC: 5.6; NET: 47.3	Localized: 45.7%; Regional: 29.9%; Distant: 24.4%	Chemotherapy: 30.5%	Median OS: 65 months (chemo); 5-year OS: 51.9%	0.58-1.63 (Increasing)	NET emergence as dominant subtype
2004-2016	Zheng et al. 2020 [32]	SEER	315	MiNEN: 100% (specialized subtype)	Localized: 27.6%; Regional: 38.7%; Distant: 33.7%	RHC: 62.2%; Appendectomy: 32.7%	5-year OS: 57.4%	0.01-0.07 (Increasing)	Novel subtype identification
2004-2014	Byrne et al. 2019 [33]	NCDB	18,055	MAC: 81.8; NMAC: 18.2; NET: 7.0 (CRS/HIPEC cohort)	Stage IV predominant: 69.1% in CRS/HIPEC group	HIPEC utilization: 7.71% overall	5-year OS: 65.6% (mucinous CRS/HIPEC)	NR	Advanced treatment option emergence
2004-2017	Marks et al. 2023 [23]	NCDB	18,216	MAC: 34.0; NMAC: 24.0; GCA: 11.0; NET: 31.0	Variable across surgical approaches	RHC: 60%; Appendectomy: 40%	Quality metrics: 30-day mortality 0.9-1.4%	NR	Quality improvement focus
2004-2019	Baron et al. 2024 [25]	NCDB	6800	MAC: 42.2; NMAC: 50.4; SRCC: 7.4	Stage I-II: 78.2%; Stage III: 21.8%	RHC: 71.5% (RM-), 59.7% (RM+); Chemotherapy: 37.3%	Median OS: 54.0 months (RM+)	NR	Surgical margin optimization
2005-2019	Emile et al. 2024 [28]	NCDB	2607	MAC: 46.0; NMAC: 45.3; SRCC: 8.7	Stage I-II: 85%; Stage III: 15%	RHC: 61.7%; Adjuvant chemotherapy: 39.4%	Median OS: 126.3 months; 5-year OS: 58.4%	NR	Contemporary outcome benchmarks
1995-2020	El Asmar et al. 2024 [24]	NCRAS; SEER	2701	GCA: 100% (goblet cell focus)	Local: 71.6% (UK), 54.8% (US); Distant: 5.7% (UK), 35.7% (US)	RHC: 71% (UK), 53% (US); Geographic variation noted	5-year OS: 73.8% (UK), 79.6% (US)	NR	International outcome comparison
**PROJECTION PERIOD**	**2030 FORECASTING MODELS**	**SCENARIO ANALYSIS**	**POPULATION SCALE**	**PREDICTED EVOLUTION**	**PROJECTED TRENDS**	**ANTICIPATED CHANGES**	**EXPECTED OUTCOMES**	**FORECASTED RATES**	**HEALTHCARE PLANNING**
2025-2030	Overall Disease Burden - Conservative	Population-based projection	25% increase in annual cases	Stable histologic distribution; MAC: 35-40%; NET: 35-40%	Localized increase to 50%; Distant decrease to 35%	RHC: 60-75%; Chemotherapy: 45-60%; HIPEC: 15-25%	5-year OS: 65-80% overall; Reduced stage variation	0.8-2.0 per 100,000 (+25% from current)	Enhanced diagnostic capacity required
	Overall Disease Burden - Optimistic	Population-based projection	50% increase in annual cases	Molecular subclassification; NET: 45%; Novel subtypes: 10%	Localized: 60%; Regional: 25%; Distant: 15%	RHC: 65-80%; Targeted therapy: 30%; HIPEC: 30-40%	5-year OS: 75-85% overall; Precision medicine benefits	1.0-2.4 per 100,000 (+50% from current)	Specialized center expansion needed
	Histologic Complexity Evolution	Pathology-based projection	Molecular characterization widespread	Precision histologic classification; Biomarker-driven subtypes	Stage migration due to improved detection	Biomarker-guided treatment selection	Subtype-specific survival optimization	Subtype-dependent incidence patterns	Advanced pathology infrastructure
	Treatment Evolution Trajectory	Technology-based projection	HIPEC center expansion	Treatment-resistant subtype identification	Earlier stage detection through screening	Minimally invasive HIPEC; Immunotherapy integration	Reduced treatment-related mortality	Treatment-specific outcome improvements	Surgical training program expansion
	Quality Metrics Improvement	Outcome-based projection	Perioperative care enhancement	Risk-stratified treatment approaches	Optimized staging protocols	Enhanced recovery protocols; Precision surgery	30-day mortality: <1%; 90-day mortality: <2%	Quality-adjusted survival gains	Survivorship program development
	Geographic Disparities Reduction	Healthcare-based projection	International standardization	Global histologic classification harmony	Uniform staging across regions	Technology transfer; Training standardization	Outcome convergence internationally	Equalized incidence detection rates	International collaboration frameworks
	Healthcare Planning Requirements	System-based projection	Population aging impact	Age-adjusted histologic patterns	Comorbidity-adjusted staging	Age-appropriate treatment algorithms	Geriatric-specific survival models	Age-stratified incidence projections	Long-term care planning integration

Abbreviations: CRS, cytoreductive surgery; GCA, goblet cell adenocarcinoma; HIPEC, hyperthermic intraperitoneal chemotherapy; LOS, length of stay; MAC, mucinous adenocarcinoma; MiNEN, mixed neuroendocrine non-neuroendocrine neoplasm; MUC, mucinous; NCDB, National Cancer Database; NCRAS, National Cancer Registration and Analysis Service; NET, neuroendocrine tumor; NMAC, non-mucinous adenocarcinoma; NON-MUC, non-mucinous; NR, not reported; OS, overall survival; PALGA, Pathologisch-Anatomisch Landelijk Geautomatiseerd Archief; RHC, right hemicolectomy; RM, resection margin; SEER, Surveillance, Epidemiology, and End Results; SRCC, signet ring cell carcinoma; UK, United Kingdom; US, United States.

Supplementary Table S3: Advanced Statistical Methodology, Validation, Uncertainty Quantification, and Evidence Quality Assessment Using Modified Newcastle-Ottawa Scale.

Study Name	Database Source	Overlap Risk Level	Overlap Assessment Justification	Data Completeness Tier	Missing Data Pattern	Validation Methods Applicable	Uncertainty Quantification Possibility
El Asmar et al. 2024 [24]	NCRAS; SEER	LOW	Minimal overlap due to distinct time period or criteria	Moderate (3/5)	Complete/Incomplete/Complete/Incomplete/Complete	Survival model validation	Confidence intervals; Survival uncertainty
Baron et al. 2024 [25]	NCDB	HIGH	NCDB database with high temporal overlap probability	High (4-5/5)	Complete/Complete/Complete/Complete/Complete	Bootstrap validation; Survival model validation; Quality metric validation; Subgroup validation	Confidence intervals; Survival uncertainty; Prediction intervals; Stratified uncertainty
Emile et al. 2024 [28]	NCDB	HIGH	NCDB database with high temporal overlap probability	High (4-5/5)	Complete/Complete/Complete/Complete/Complete	Survival model validation; Quality metric validation; Subgroup validation	Confidence intervals; Survival uncertainty; Prediction intervals; Stratified uncertainty
Freudenberger et al. 2023 [29]	NCDB	HIGH	NCDB database with high temporal overlap probability	Moderate (3/5)	Complete/Incomplete/Incomplete/Complete/Complete	Survival model validation; Quality metric validation	Confidence intervals; Survival uncertainty; Prediction intervals
Marks et al. 2023 [23]	NCDB	MEDIUM	NCDB database with moderate temporal overlap	Moderate (3/5)	Incomplete/Complete/Complete/Complete/Incomplete	Bootstrap validation; Quality metric validation; Subgroup validation	Confidence intervals; Prediction intervals; Stratified uncertainty
Wang et al. 2023 [30]	SEER	MEDIUM	SEER database with moderate temporal overlap	High (4-5/5)	Complete/Complete/Complete/Incomplete/Complete	Survival model validation; Subgroup validation	Confidence intervals; Survival uncertainty; Stratified uncertainty
Wang et al. 2021 [31]	SEER	MEDIUM	SEER database with moderate temporal overlap	High (4-5/5)	Complete/Complete/Complete/Incomplete/Complete	Bootstrap validation; Survival model validation; Subgroup validation	Confidence intervals; Survival uncertainty; Stratified uncertainty
Zheng et al. 2020 [32]	SEER	LOW	Minimal overlap due to distinct time period or criteria	Moderate (3/5)	Complete/Incomplete/Complete/Incomplete/Complete	Survival model validation	Survival uncertainty
Byrne et al. 2019 [33]	NCDB	HIGH	NCDB database with high temporal overlap probability	Moderate (3/5)	Complete/Complete/Incomplete/Incomplete/Complete	Bootstrap validation; Survival model validation; Subgroup validation	Confidence intervals; Survival uncertainty; Stratified uncertainty
Yan et al. 2019 [34]	SEER	MEDIUM	SEER database with moderate temporal overlap	High (4-5/5)	Complete/Complete/Complete/Incomplete/Complete	Survival model validation; Subgroup validation	Confidence intervals; Survival uncertainty; Stratified uncertainty
Shaib et al. 2017 [35]	SEER	MEDIUM	SEER database with moderate temporal overlap	High (4-5/5)	Complete/Complete/Complete/Incomplete/Complete	Survival model validation; Subgroup validation	Confidence intervals; Survival uncertainty; Stratified uncertainty
Xie et al. 2016 [21]	SEER	LOW	Minimal overlap due to distinct time period or criteria	High (4-5/5)	Complete/Complete/Complete/Incomplete/Complete	Survival model validation; Subgroup validation	Confidence intervals; Survival uncertainty; Stratified uncertainty
Asare et al. 2016 [36]	NCDB	MEDIUM	NCDB database with moderate temporal overlap	High (4-5/5)	Complete/Complete/Complete/Incomplete/Complete	Bootstrap validation; Survival model validation; Subgroup validation	Confidence intervals; Survival uncertainty; Stratified uncertainty
Ansari et al. 2016 [37]	Institutional	NONE	No overlap - unique database or institution	Moderate (3/5)	Complete/Incomplete/Incomplete/Complete/Complete	Survival model validation; Quality metric validation	Confidence intervals; Survival uncertainty; Prediction intervals
Marmor et al. 2015 [13]	SEER	HIGH	SEER database with high temporal overlap probability	Moderate (3/5)	Complete/Complete/Incomplete/Incomplete/Complete	Survival model validation; Subgroup validation	Confidence intervals; Survival uncertainty; Stratified uncertainty
Turaga et al. 2012 [38]	SEER	HIGH	SEER database with high temporal overlap probability	High (4-5/5)	Complete/Complete/Complete/Incomplete/Complete	Bootstrap validation; Survival model validation; Subgroup validation	Confidence intervals; Survival uncertainty; Stratified uncertainty
Smeenk et al. 2008 [39]	PALGA	NONE	No overlap - unique database or institution	Limited (≤2/5)	Complete/Complete/Incomplete/Incomplete/Incomplete	Subgroup validation	Confidence intervals; Stratified uncertainty
McCusker et al. 2002 [40]	SEER	HIGH	SEER database with high temporal overlap probability	Moderate (3/5)	Complete/Complete/Complete/Incomplete/Incomplete	Subgroup validation	Confidence intervals; Stratified uncertainty

Abbreviations: NCDB, National Cancer Database; NCRAS, National Cancer Registration and Analysis Service; PALGA, Pathologisch-Anatomisch Landelijk Geautomatiseerd Archief; SEER, Surveillance, Epidemiology, and End Results; Demo, demographics; Histo, histology; Treat, treatment; Complete, data available and reported; Incomplete, data not reported or insufficient detail; Bootstrap, bootstrap resampling validation; Subgroup, subtype-specific validation; Quality metric, perioperative outcome validation; Survival model, time-to-event model validation; Confidence intervals, parameter uncertainty quantification; Prediction intervals, individual prediction uncertainty; Survival uncertainty, time-to-event uncertainty quantification; Stratified uncertainty, subgroup-specific uncertainty analysis.
